# Chromosome-scale genome assembly of *Prunus pusilliflora* provides novel insights into genome evolution, disease resistance, and dormancy release in *Cerasus* L.

**DOI:** 10.1093/hr/uhad062

**Published:** 2023-04-10

**Authors:** Songtao Jiu, Baozheng Chen, Xiao Dong, Zhengxin Lv, Yuxuan Wang, Chunjin Yin, Yan Xu, Sen Zhang, Jijun Zhu, Jiyuan Wang, Xunju Liu, Wanxia Sun, Guoqian Yang, Meng Li, Shufeng Li, Zhuo Zhang, Ruie Liu, Lei Wang, Muhammad Aamir Manzoor, Quero-García José, Shiping Wang, Yahui Lei, Ling Yang, Elisabeth Dirlewanger, Yang Dong, Caixi Zhang

**Affiliations:** Department of Plant Science, School of Agriculture and Biology, Shanghai Jiao Tong University, Shanghai, 200240, P. R. China; College of Food Science and Technology, Yunnan Agricultural University, Kunming, Yunnan Province, 650201, P. R. China; College of Food Science and Technology, Yunnan Agricultural University, Kunming, Yunnan Province, 650201, P. R. China; Department of Plant Science, School of Agriculture and Biology, Shanghai Jiao Tong University, Shanghai, 200240, P. R. China; Department of Plant Science, School of Agriculture and Biology, Shanghai Jiao Tong University, Shanghai, 200240, P. R. China; Dali Bai Autonomous Prefecture Academy of Agricultural Sciences and Extension, Dali, Yunnan Province, 671600, P. R. China; Department of Plant Science, School of Agriculture and Biology, Shanghai Jiao Tong University, Shanghai, 200240, P. R. China; Department of Plant Science, School of Agriculture and Biology, Shanghai Jiao Tong University, Shanghai, 200240, P. R. China; Shanghai Botanical Garden, Shanghai, 200231, P. R. China; Department of Plant Science, School of Agriculture and Biology, Shanghai Jiao Tong University, Shanghai, 200240, P. R. China; Department of Plant Science, School of Agriculture and Biology, Shanghai Jiao Tong University, Shanghai, 200240, P. R. China; Department of Plant Science, School of Agriculture and Biology, Shanghai Jiao Tong University, Shanghai, 200240, P. R. China; Department of Plant Science, School of Agriculture and Biology, Shanghai Jiao Tong University, Shanghai, 200240, P. R. China; College of Biology and the Environment, Nanjing Forestry University, Nanjing, Jiangsu Province, 200037, P. R. China; Dali Bai Autonomous Prefecture Academy of Agricultural Sciences and Extension, Dali, Yunnan Province, 671600, P. R. China; Department of Plant Science, School of Agriculture and Biology, Shanghai Jiao Tong University, Shanghai, 200240, P. R. China; Department of Plant Science, School of Agriculture and Biology, Shanghai Jiao Tong University, Shanghai, 200240, P. R. China; Department of Plant Science, School of Agriculture and Biology, Shanghai Jiao Tong University, Shanghai, 200240, P. R. China; Department of Plant Science, School of Agriculture and Biology, Shanghai Jiao Tong University, Shanghai, 200240, P. R. China; INRAe, UMR 1332 de Biologie du Fruit et Pathologie, 33140 Villenave d'Ornon, France; Department of Plant Science, School of Agriculture and Biology, Shanghai Jiao Tong University, Shanghai, 200240, P. R. China; College of Food Science and Technology, Yunnan Agricultural University, Kunming, Yunnan Province, 650201, P. R. China; College of Food Science and Technology, Yunnan Agricultural University, Kunming, Yunnan Province, 650201, P. R. China; INRAe, UMR 1332 de Biologie du Fruit et Pathologie, 33140 Villenave d'Ornon, France; College of Food Science and Technology, Yunnan Agricultural University, Kunming, Yunnan Province, 650201, P. R. China; Department of Plant Science, School of Agriculture and Biology, Shanghai Jiao Tong University, Shanghai, 200240, P. R. China

## Abstract

*Prunus pusilliflora* is a wild cherry germplasm resource distributed mainly in Southwest China. Despite its ornamental and economic value, a high-quality assembled *P. pusilliflora* genome is unavailable, hindering our understanding of its genetic background, population diversity, and evolutionary processes. Here, we *de novo* assembled a chromosome-scale *P. pusilliflora* genome using Oxford Nanopore, Illumina, and chromosome conformation capture sequencing. The assembled genome size was 309.62 Mb, with 76 scaffolds anchored to eight pseudochromosomes. We predicted 33 035 protein-coding genes, functionally annotated 98.27% of them, and identified repetitive sequences covering 49.08% of the genome. We found that *P. pusilliflora* is closely related to *Prunus serrulata* and *Prunus yedoensis*, having diverged from them ~41.8 million years ago. A comparative genomic analysis revealed that *P. pusilliflora* has 643 expanded and 1128 contracted gene families. Furthermore, we found that *P. pusilliflora* is more resistant to *Colletotrichum viniferum*, *Phytophthora capsici*, and *Pseudomonas syringae* pv. *tomato* (*Pst*) DC3000 infections than cultivated *Prunus avium*. *P. pusilliflora* also has considerably more nucleotide-binding site-type resistance gene analogs than *P. avium*, which explains its stronger disease resistance. The cytochrome P450 and WRKY families of 263 and 61 proteins were divided into 42 and 8 subfamilies respectively in *P. pusilliflora*. Furthermore, 81 MADS-box genes were identified in *P. pusilliflora*, accompanying expansions of the SVP and AGL15 subfamilies and loss of the TM3 subfamily. Our assembly of a high-quality *P. pusilliflora* genome will be valuable for further research on cherries and molecular breeding.

## Introduction

**Figure 1:**
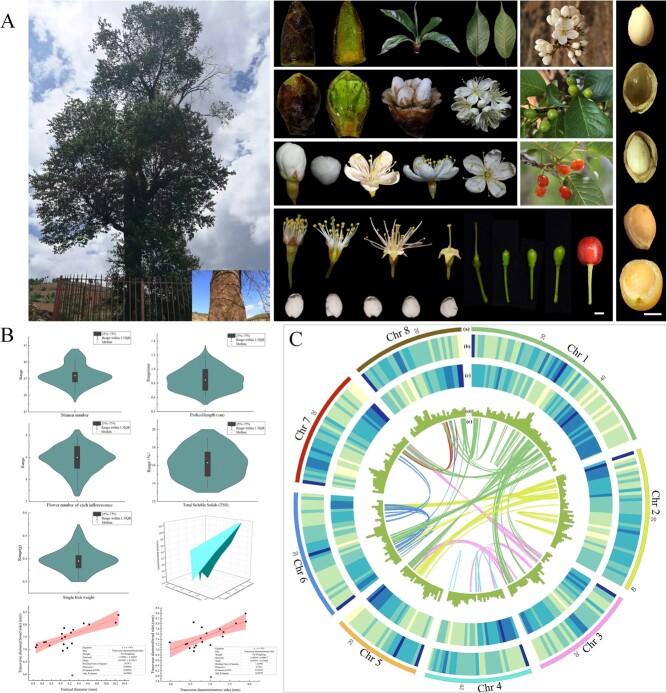
*De novo* genome assembly of *P. pusilliflora*. (**A**) Phenotypic characteristics of *P. pusilliflora* flowers, buds, leaves, berries, and seeds collected between February and May 2022. Scale bar = 2 cm. (**B**) Parameters of stamens and flowers, pedicel length, total soluble solids, vertical and transverse diameter, and single fruit weight. (**C**) Summary of *de novo* genome assembly and sequencing analysis of *P. pusilliflora.* (a) Chromosome size (Mb). (b) Repeat and (c) gene density in 1-Mb sliding windows. (d) GC content in 1-Mb sliding windows, and (e) synteny blocks among *P. pusilliflora* chromosomes.

The Rosaceae family consists of ~3000 species, distributed across 90 genera with abundant fruit types [1]. It contains most of the temperate fruit species categorized as stone and pome fruits depending on their fruit morphology, with fleshy fruits that are abundant in organic acids, carbohydrates, vitamins, carotene, and minerals. The stone fruit *Prunus pusilliflora* (Ppus) Card. belongs to the subgenus *Cerasus* in the Rosaceae family and might be the parent of several flowering and fresh Chinese cherry germplasm resources. Widely distributed in Yunnan and Sichuan provinces of Southwest China, endemic Ppus is a wild woody plant that grows naturally on the sides of ravines and sunny mountain slopes at altitudes of 1400–2600 m. The plant has dark-green leaves with acuminate serrate teeth, corymbose-racemose inflorescences with three to seven flowers, white single suborbicular petals, 33–43 stamens nearly as long as its petals, and purple-black fruits (Fig. 1A and B; Supplementary Data Table S1). Ppus has excellent horticultural characteristics, such as vigorous growth, a robust root system, plasticity, and stress tolerance, highlighting its potential as a rootstock. Furthermore, the ornamental and lumber value of this species, increasing its economic importance, makes Ppus worthy of further investigation.

China has the most abundant wild cherry germplasm resources worldwide, resulting in interspecific or intergeneric hybrids and the breeding of numerous varieties [2, 3]. Due to a long history of frequent cultivation and hybridization in addition to natural selection, some taxonomic controversies often arise concerning the exact names, origins, and definitions of different populations of cherry germplasms, especially in the wild. One of the scientific issues is the genetic relationships among Ppus, *Prunus yedoensis* (Pyed), and *Prunus serrulata* (Pser). Insufficient systematic classification and biological evidence have generated confusion regarding the taxonomic groups of Ppus and other *Cerasus* species. Complicating matters, few investigations have been launched on Ppus, resulting in a lack of specimen collections, mining and utilization of morphological and molecular markers, and phylogenetic analyses. Considering that genomic studies have contributed to solving these issues to a certain extent, we conducted *de novo* genome assembly of Ppus, with the aim of providing a scientific basis for investigating the evolutionary processes in this species.

Commercial cherry production is confronted with many challenges from biotic and abiotic stresses. Bacterial canker caused by *Pseudomonas syringae* is one of the most devastating diseases in cherries [4, 5], having caused tremendous losses in the global yield of cherries [6–8]. Other microorganisms, such as *Phytophthora*, *Colletotrichum*, and *Botrytis cinerea*, inflict diseases that limit cherry productivity [9–12]. Most plant resistance genes (R genes) belong to the nucleotide-binding site leucine-rich repeat (NLR) receptor family, which confers resistance to various pathogens, including bacteria, viruses, oomycetes, and fungi [13, 14]. The WRKY gene family also plays crucial roles in pathogen defense and environmental stress responses [15–17]. The cytochrome P450 monooxygenases (CYP450), a family of heme-thioate proteins, protect plants from diseases and insect infestations [18, 19]. Therefore, a major objective of cherry breeding programs worldwide is to improve disease resistance and the abiotic tolerance of cultivated cherries by investigating the resistance and tolerance genes.

Bud dormancy, a complex process comprising many biological events, is essential for cherry growth and development. Its release is triggered by long-term exposure to cold, and cold accumulation in winter is commonly addressed as the chilling requirement. Global warming has led to inadequate chilling accumulation in winter, which has caused physiological disorders along with some negative effects on flowering, bud sprouting, and fruit production [20]. The *Dormancy-Associated MADS-Box* (*DAM*) genes, belonging to the SHORT VEGETATIVE PHASE (SVP)/AGAMOUS 24 (AGL24) subfamily of the MADS-box family, are involved in dormancy regulation [20, 21]. The large fragment deletion involving *DAM1–4*, which also eliminates *DAM5* and *DAM6* expression, stops bud growth cessation in the *evergrowing* (*evg*) peach mutant [22]. Therefore, elucidating the mechanism controlling dormancy release that involves MADS-box family members might help address some issues caused by climate change.

The combined use of Oxford Nanopore Technologies (ONT) sequencing, next-generation sequencing (NGS), and chromosome conformation capture (Hi-C) sequencing has been particularly fruitful for genome assembly [23–25]. Owing to the highly heterozygous genetic background of *Cerasus* species, their genome sequencing and assembly are challenging. Nevertheless, high-quality genome assemblies have contributed to clarifying the phylogenetic relationships and resolving taxonomic controversies in this subgenus. In fact, whole genomes have been sequenced in various *Prunus* crops, including *P. avium* (Pavi) [3], Pser [26], Pyed [27], *P. fruticosa* [28], *P. dulcis* (Pdul) [29], *P. domestica* [30], *P. salicina* [31], and *P. mume* [32]. As such data were previously unavailable for Ppus, we therefore generated a high-quality chromosome-level genome assembly. We then compared the Ppus genome with the publicly available *Cerasus* L. genomes and investigated gene family evolution, positive selection, and disease resistance in Ppus. This study provides a solid foundation for elucidating the genetic diversity, variation, phylogenic hierarchy, and mechanism underlying the strong disease resistance of Ppus. The sequenced genome will be a valuable resource for basic research on cherries and molecular breeding.

## Results

### Genome sequencing and assembly

The Ppus genome is estimated to be 303.03 Mb based on *k*-mer frequencies of Illumina short reads (Supplementary Data Table S2). We generated the Ppus genome by integrating NGS, ONT, and Hi-C sequencing. We obtained 93.35 Gb (~301.5×) of ONT clean data, 67.72 Gb (~218.7×) of Illumina clean data, and 87.88 Gb (~283.8×) of Hi-C data (Supplementary Data Table S3). The assembled Ppus genome was 309.53 Mb, containing 265 contigs after assembly with long reads and correction with short reads. The chromosome-level genome assembly contained 76 scaffolds, covering a total size of 309.62 Mb, with a GC content of 38.02% (Supplementary Data Table S4). Of the scaffolds, 290 228 734 bp were anchored to eight pseudochromosomes and covered ~93.74% of the assembly (Fig. 1C; Supplementary Data Table S5). The genome size of Ppus resembled that of Pyed var. *nudiflora* [27], was larger than that of Pser [26], and was smaller than that of Pavi cv. Tieton [3] (Table 1). A Hi-C interaction heat map indicated that the Ppus genome had no obvious assembly errors and comprised eight clusters at the chromosomal level (Supplementary Data Fig. S1). To evaluate genome quality and completeness, Illumina paired-end short reads were aligned to the final assembled genome using the Burrows–Wheeler Alignment-Maximal Exact Match (BWA-MEM) software. Approximately 96.73% of the reads mapped to the assembly (Supplementary Data Table S6). Benchmarking Universal Single-Copy Orthologs (BUSCO) analysis of the final assembly indicated 98.30% completeness, with only 1.70% missing single-copy orthologs (Supplementary Data Table S7). In addition, as the long terminal repeat (LTR) assembly index (LAI) is often used to evaluate the quality of a genome assembly, we compared the LAI of Ppus with those of several Rosaceae species. We found that the LAI of Ppus (17.35) was only slightly lower than that of the well-assembled Pavi (19.68) but higher than the LAI of *Prunus armeniaca* (Parm) (16.29), Pyed (6.87), and *P. domestica* (2.27), indicating that it has a superior assembly quality (Supplementary Data Table S8).

**Table 1:** Comparison of the *P. pusilliflora* genome with previously published assemblies of the genus *Cerasus* genome.

**Category**	***Prunus pusilliflora***	***Prunus avium* cv. Tieton**	***Prunus serrulata***	***Prunus yedoensis* var. *nudiflora***
Total assembly size	309.62 Mb	344 287 078 bp	265.4 Mb	318.7 Mb
Complete BUSCOs number	1587	1525	1528	
Complete BUSCOs (%)	98.3	97.4	94.6	
Contig N50	6 002 992 bp	3247.20 kb	1.56 Mb	132 585 bp
Longest contig	24 643 276 bp	13603.98 kb	7.34 Mb	773 088 bp
Total scaffold number	76		67	3185
Scaffold N50	33 870 278 bp		31.12 Mb	198 954 bp
Longest scaffold	59 339 823 bp		49.87 Mb	960 226 bp
GC content (%)	38.6	38.44	38.51	
Number of protein-coding genes	33 035	40 338	29 094	41 294
Repeat content (%)	49.08		49.02	

### Gene prediction and annotation

We identified 142.44 Mb repetitive sequences (~49.08% of the genome) including simple repeats, and transposable elements (Supplementary Data Table S9). Among these repetitive sequences, ~121.25 Mb (~41.79% of the genome) were classified into different types of transposable elements (Supplementary Data Table S10). The repeat-masked genome was used as input data for gene predictors. We annotated 33035 protein-coding genes in Ppus, supported by homologous and *de novo* predictions (Supplementary Data Table S11). The BUSCO completeness between the genome (98.30%) and the annotated gene set (96.2%) was close, indicating the successful annotation of most genes in the Ppus genome (Supplementary Data Table S7). We functionally annotated 32463 genes using the non-redundant (NR) (32453 genes), eggNOG (27458), Swiss-Prot (22172), Pfam (21905), Clusters of Orthologous Groups of proteins (COG) (27458), Gene Ontology (GO) (10174), and Kyoto Encyclopedia of Genes and Genomes (KEGG) (12888) databases (Supplementary Data Tables S12 and S13). We also identified 149 micro-, 756 transfer, 1247 ribosomal, and 276 small nuclear (sn) RNAs in the Ppus genome (Supplementary Data Table S14).

### Syntenic analysis between *P. pusilliflora* and three *Prunus* species

**Figure 2:**
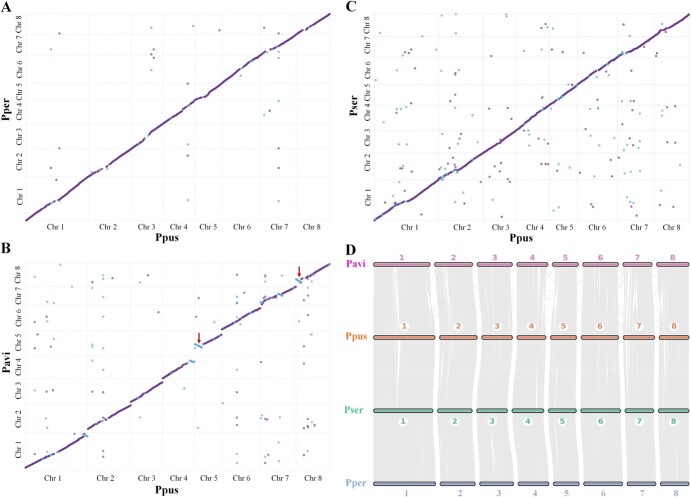
Synteny analysis of *P. pusilliflora*, *P. persica*, *P. serrulata*, and *P. avium*. Synteny map of the *P. pusilliflora* genome and the (**A**) *P. persica*, (**B**) *P. avium*, and (**C**) *P. serrulata* genomes. Purple and blue denote similar sequences in the same and opposite orientations, respectively. (**D**) Syntenic blocks among *P. pusilliflora*, *P. persica*, *P. serrulata*, and *P. avium*. Numbers represent the chromosome order from the original genome sequence. Each line represents one block. Red arrows indicate chromosomal inversion.

To gain a better understanding of the relationship between Ppus and several *Prunus* species, we performed a syntenic analysis and drew synteny maps after comparing the Ppus genome with *Prunus persica* (Pper), Pavi, and Pser genomes (Fig. 2A–C). The Ppus versus Pser synteny map showed a strong collinear relationship with 3778 syntenic blocks, of which 3645 and 133 were located on homologous and different chromosomes, respectively (Supplementary Data Table S15). Furthermore, the statistical results of the Ppus versus Pavi synteny map revealed 2722 and 81 syntenic blocks on homologous and different chromosomes, respectively (Supplementary Data Table S16). The Ppus versus Pper synteny map showed 2552 and 24 syntenic blocks on homologous and different chromosomes, respectively (Supplementary Data Table S17). The ratio (268/2803) of homologous sequences within the Ppus versus Pavi synteny map in opposite orientations, as indicated by blue dots in Fig. 2, was higher than the ratios of Ppus versus Pser (167/3611) and Ppus versus Pper (36/2542) synteny maps (Supplementary Data Table S18). Meanwhile, several chromosome inversions were present in the Ppus versus Pavi synteny map (Fig. 2B). Gene syntenic blocks derived from comparing the Ppus genome with the Pper, Pavi, and Pser genomes were distributed across eight chromosomes, showing a strong cross-species synteny (Fig. 2D). We observed 149 gene syntenic blocks consisting of 34762 genes between Ppus and Pavi; 98 of these blocks were located on the same chromosome (Supplementary Data Table S19). Furthermore, 68, 60, and 34 blocks were identified when comparing Ppus and Pser, Ppus and Pper, and Pser and Pper on the same chromosome, respectively (Supplementary Data Tables S20–S22). All syntenic blocks were accurately matched on the same chromosome between each of the three pairs (Supplementary Data Tables S20–S22).

### Evolution and gene family expansion analysis

**Figure 3:**
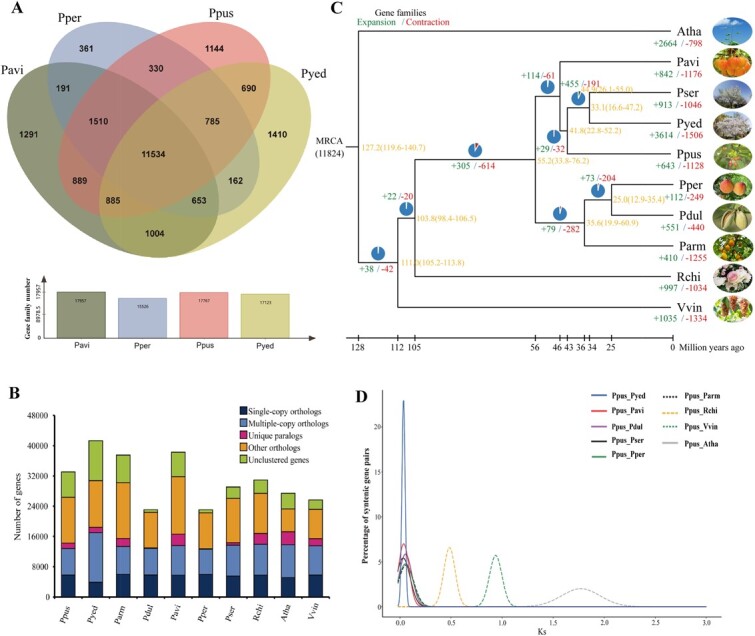
Comparative analysis of gene families between the genomes of *P. pusilliflora* and other species. (**A**) Venn diagram showing shared and unique gene families among four sequenced genomes of *Prunus*. (**B**) Gene number distribution of single-copy, multiple-copy, and other orthologs as well as unique paralogs, and unclustered genes. (**C**) Phylogenetic tree, divergence time, and profiles of gene families that underwent contraction, expansion, and rapid evolution in 10 species. (**D**) *K*_s_ distribution of orthologous gene pairs from *P. pusilliflora* (Ppus) compared with *A. thaliana* (Atha), *P. avium* (Pavi), *P. serrulata* (Pser), *P. persica* (Pper), *P. yedoensis* (Pyed), *P. dulcis* (Pdul), *P. armeniaca* (Parm), *R. chinensis* (Rchi), and *V. vinifera* (Vvin).

Orthologous clustering was conducted on the Ppus, Pavi, Pper, and Pyed genomes (Supplementary Data Tables S23–S26). We identified 17 767 gene families in the Ppus genome, which was more than the number in the Pper and Pyed genomes and slightly less than that in the Pavi genome (Fig. 3A). Moreover, 11 534 gene families were common to all four *Prunus* plants, whereas more unique gene families (1144) were found in the Ppus than in the Pper genome (Fig. 3A). To elucidate evolutionary relationships, we performed a comparative genomic analysis of all the identified families based on our BLASTP and Pfam results (Supplementary Data Table S27). We then compared the numbers of single- and multiple-copy orthologs, other orthologs, unique paralogs, and unclustered genes among *Arabidopsis thaliana* (Atha), Pavi, Pser, Pyed, Ppus, Pper, Pdul, Parm, *Rosa chinensis* (Rchi), and *Vitis vinifera* (Vvin), and selected 1938 high-quality single-copy orthologs for phylogenetic reconstruction (Fig. 3B). Detailed statistics of unique, expanded, and contracted gene families in the Ppus genome are shown in Supplementary Data Tables S28–S30. Among 11 824 gene families common to the 10 species, 643 and 1128 gene families expanded and contracted, respectively, in Ppus after speciation from Pavi (Fig. 3C). Ppus contained fewer expanded gene families than did Pavi, Pser, and Pyed (Fig. 3C). However, the number of contracted gene families was greater in Ppus than in Pser. The expanded, contracted, and unique family genes were significantly enriched (*P* < 0.05) in 37, 102, and 109 GO terms, respectively (Supplementary Data Tables S31–S33). Expanded gene families were the most significantly enriched in terms of ‘ADP binding’ in molecular function (MF), ‘signaling’ in biological process (BP), and ‘intrinsic component of membrane’ in cellular component (Supplementary Data Fig. S2A; Supplementary Data Table S31). Contracted gene families were the most significantly enriched in terms of ‘phosphorus metabolic process’ in BP, ‘nucleoside phosphate binding’ in MF, and ‘membrane’ in cellular component (Supplementary Data Fig. S2B; Supplementary Data Table S32). Unique gene families were the most significantly enriched in terms of ‘cysteine-type peptidase activity’ in MF, ‘proteolysis’ in BP, and ‘proton-transporting two-sector ATPase complex, catalytic domain’ in cellular component (Supplementary Data Fig. S2C; Supplementary Data Table S33).

### Comparative genomic analysis of *P. pusilliflora*

We assessed the divergence times of Ppus and nine other species based on the phylogenetic tree (Fig. 3C). Bayesian molecular dating analysis showed that the genera *Prunus* and *Rosa* diverged around 103.8 million years ago (Mya) [95% highest posterior densities (HPDs) of 98.4–106.5 Mya], whereas the subgenera *Armeniaca*/*Amygdalus* and *Cerasus* diverged around 55.2 Mya (95% HPD of 33.8–76.2 Mya). The divergence time between the subgenera *Prunus* (Parm) and *Amygdalus* (Pper and Pdul) was ~35.6 Mya (95% HPD of 19.9–60.9 Mya). Similarly, the split between Pavi and Ppus occurred ~44.9 Mya (95% HPD of 26.1–55.0 Mya). The phylogenetic tree indicated that Ppus was most closely associated with two flowering cherries (Pser and Pyed), having diverged from them ~41.8 Mya (95% HPD of 22.8–52.2 Mya). Homologous genes obtained via the Nei–Gojobori (NG86) method were subjected to the Ks Calculator for gene pair estimation [33]. The numbers of positively selected orthologous gene pairs (*K*_a_/*K*_s_ > 1) for Ppus versus Pavi and for Pser versus Ppus were 1080 and 1151, respectively (Supplementary Data Tables S34 and S35). Transcription factors (TFs) with definitively matched Pfam domains were identified from these gene pairs, resulting in 59 positively selected TF genes for Ppus versus Pavi and 54 for Ppus versus Pser (Supplementary Data Tables S36 and S37). Functional analyses of common TFs (e.g. MADS, WRKY, MYB, ERF, bZIP, NAC, and bHLH) indicated that they are more likely to participate in growth and development, stress response, and physiological metabolism in Ppus. After comparing the synonymous substitution rates (*K*_s_), we determined that Ppus had experienced one whole-genome duplication (WGD) event, similar to Pper, Parm, Pser, and Pdul (Supplementary Data Table S38). Divergence of the Ppus genome from nine angiosperm genomes was deduced based on *K*_s_ distributions of orthologous genes (Fig. 3D). Ppus exhibited a single peak with Pser, Pyed, Pavi, Pper, Pdul, and Pram at *K*_s_ values of 0.0320, 0.0326, 0.0368, 0.0545, 0.0548, and 0.0602, respectively (Supplementary Data Fig. S3; Supplementary Data Table S39), indicating successive splitting into the subgenera *Cerasus* (Pser, Pyed, and Pavi), *Amygdalus* (Pper and Pdul), and *Armeniaca* (Pram). Our results showed that Pavi diverged earlier than Ppus, Pser, and Pyed (Supplementary Data Fig. S3). The *K*_s_ distribution among the seven *Prunus* genomes revealed that these species diversified very recently.

### *P. pusilliflora* was more resistant to *Colletotrichum viniferum*, *Phytophthora capsici*, and *Pseudomonas syringae* pv. *tomato* DC3000 than *P. avium*

**Figure 4:**
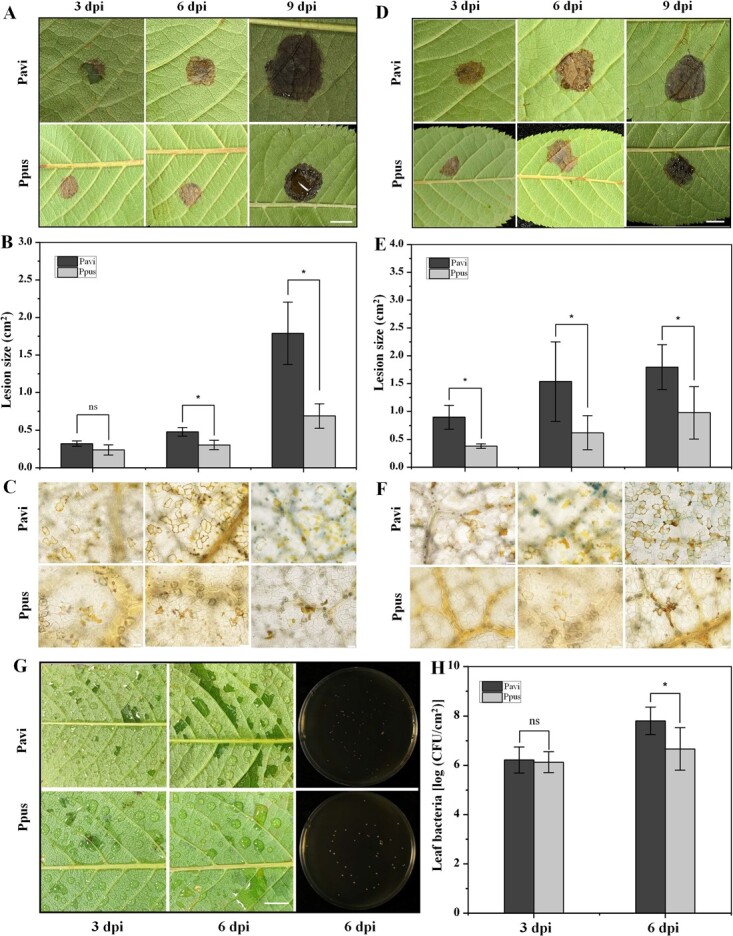
Evaluation of resistance in *P. pusilliflora* and *P. avium* leaves inoculated with pathogens. Lesions were photographed and quantified at 3, 6 and 9 dpi with *C. viniferum* (**A**, **B**) and *P. capsici* (**D**, **E**). Leaves were stained with lactophenol TB at 3, 6, and 9 dpi with *C. viniferum* (**C**) and *P. capsici* (**F**), and then areas ~1 mm from the lesions were photographed using a BX43 microscope. (**G**) Leaves were photographed at 3 and 6 dpi with *Pst* DC3000. (**H**) Bacterial proliferation was determined at 3 and 6 dpi as CFU/cm^2^ leaf. Data are shown as the mean ± standard deviation of values from each independent experiment with more than nine replicates. ^*^P < 0.05 (two-tailed Student’s *t* test); ns, not significant. Scale bars, 0.5 cm.

We evaluated the disease resistance and susceptibility of Ppus and Pavi leaves inoculated with *Colletotrichum viniferum*, *Phytophthora capsici*, *Pseudomonas syringae* pv. *tomato* (*Pst*) DC3000, and *B. cinerea* (Fig. 4; Supplementary Data Fig. S5). Symptoms were monitored every 3 days. An obvious lesion area persisted on all Ppus and Pavi leaves infected with *C. viniferum* (Fig. 4A). ImageJ measurements showed a larger lesion area in infected Pavi than in Ppus leaves at 6 and 9 days post-inoculation (dpi) (Fig. 4B). Lactophenol Trypan Blue (TB) staining identified more necrotic cells in Pavi leaves infected with *C. viniferum* than in Ppus leaves (Fig. 4C). In addition, data showed that the ratio of pathogen DNA to plant DNA increased continuously from 1 to 6 dpi, indicating *C. viniferum* could infect Ppus and Pavi (Supplementary Data Fig. S4A). Meanwhile, a significantly greater ratio was observed in Pavi than in Ppus at 6 dpi, indicating that Ppus was more resistant to *C. viniferum* fungus than Pavi*.* Similarly, the lesions on Ppus and Pavi leaves infected with *P. capsici* oomycetes increased continuously from 3 to 9 dpi but were larger on infected Pavi leaves (Fig. 4D and E). Lactophenol TB staining identified more necrotic cells in Pavi leaves than in Ppus leaves (Fig. 4F). Data revealed that the ratio of pathogen to plant DNA increased continuously from 1 to 9 dpi and showed a greater ratio in Pavi than in Ppus (Supplementary Data Fig. S4B), suggesting that *P. capsici* can infect two tested species, and Ppus is more resistant to *P. capsici* than Pavi.

*P. syringae* pv. *tomato* is a vital model pathogen for plant–pathogen interactions [34]. This pathogen caused less severe disease symptoms (water-soaking) in Ppus compared with those in Pavi leaves (Fig. 4G). We investigated whether differences in water-soaking size and disease severity in Ppus and Pavi leaves mirrored the differences in bacterial growth by counting bacteria numbers in the leaves of Ppus and Pavi at 3 and 6 dpi. Data revealed a slightly lower bacterial number in Ppus than in Pavi leaves at 3 dpi but this difference was not obvious in either. The bacterial number in Pavi leaves reached a maximum of 6.5 × 10^7^ colony-forming units (CFU)/cm^2^ at 6 dpi, which exceeded that in Ppus leaves (Fig. 4H). Meanwhile, the ratio of pathogen to plant DNA increased continuously from 3 to 6 dpi and was significantly higher in Pavi than in Ppus at 3 and 6 dpi (Supplementary Data Fig. S4C), indicating that *Pst* DC3000 can infect two tested species, Ppus and Pavi, and Ppus is more resistant to *Pst* DC3000 than Pavi. In addition, lesion size was larger in Ppus than in Pavi leaves infected with *B. cinerea* at 3 and 6 dpi (Supplementary Data Fig. S5). Finally, lactophenol TB staining identified more necrotic cells in Ppus leaves than in Pavi leaves (Supplementary Data Fig. S5). These results showed that Ppus is less resistant to the fungus *B. cinerea* than Pavi. These data together suggested that Ppus was more resistant to *C. viniferum*, *P. capsici*, and *Pst* DC3000 than cultivated Pavi, which might be associated with the natural selection of Ppus in the wild.

### Identification and analysis of NLR gene families in *P. pusilliflora*

**Figure 5:**
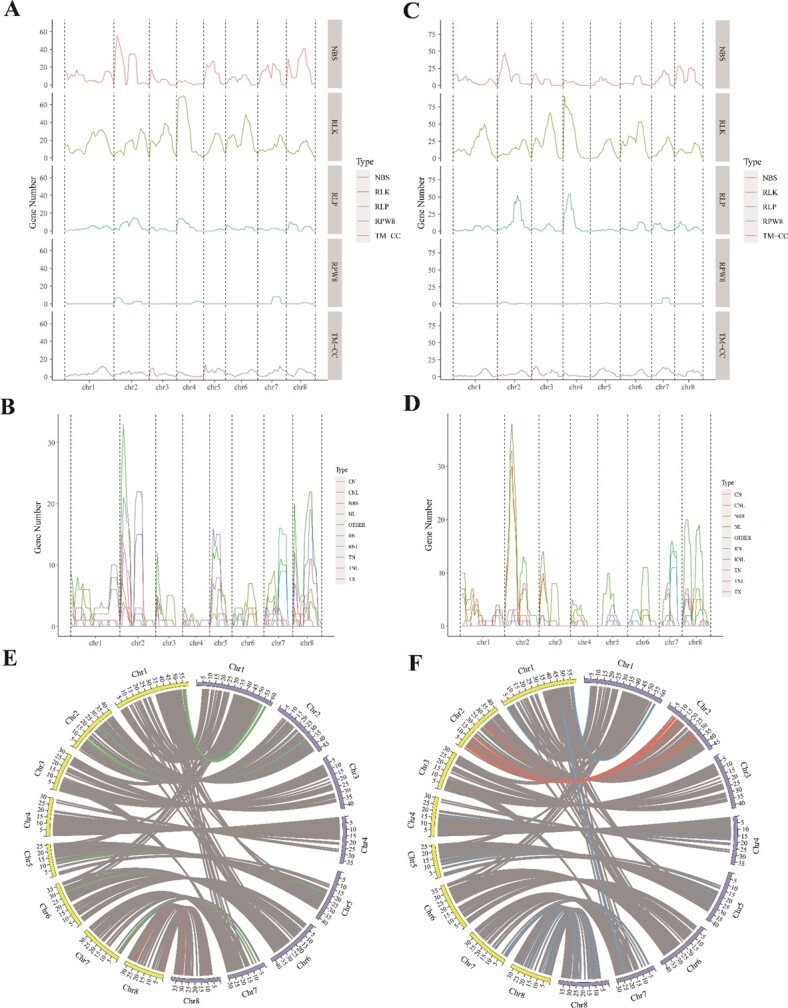
Distribution of RGAs in *P. pusilliflora* (Ppus) and *P. avium* (Pavi) chromosomes. Distribution of RGAs along *P. pusilliflora* (**A**) and *P. avium* (**C**) chromosomes, showing the absolute number of genes homologous to nucleotide-binding site–leucine-rich repeat (NBS-LRR-encoding) proteins, RLKs, RLPs, resistance to powdery mildew 8 (RPW8), and TM-CC proteins along each of the eight chromosomes. Distribution of NBS-LRR-encoding proteins along the *P. pusilliflora* (**B**) and *P. avium* (**D**) chromosomes. (**E**) Microsynteny analysis of TNL-type genes between *P. pusilliflora* and *P. avium* chromosomes, as indicated by red (representing collinear gene pairs on Chr 8) and green curves, respectively. (**F**) Microsynteny analysis of NL-type genes between the *P. pusilliflora* and *P. avium* chromosomes, as indicated by red (representing collinear gene pairs on Chr 2) and blue curves, respectively. The yellow and purple curves represent Chr1–Chr8 of *P. pusilliflora* and *P. avium*, respectively*.*

To further investigate the differences in resistance to disease resistance between Ppus and Pavi, we conducted a genome-wide analysis of resistance gene analogs (RGAs). Using a pipeline for the genome-wide prediction of RGAs [35], we identified 1330 RGA proteins in Ppus, with 1235 RGAs on eight chromosomes and 95 RGAs on unplaced scaffolds (Supplementary Data Table S40). We divided R genes into five major types based on their toll-interleukin-1 receptor (TIR)/resistance to powdery mildew 8 (RPW8)/leucine-rich repeat (LRR) and coiled-coil (CC) domains: nucleotide-binding site (NBS)-encoding proteins; receptor-like proteins (RLPs); receptor-like protein kinases (RLKs); RPW8; and transmembrane coiled-coil proteins (TM-CCs). We identified 404 NBS-type, 632 RLK-type, 126 RLP-type, 23 RPW8-type, and 145 TM-CC-type proteins in Ppus (Fig. 5A; Supplementary Data Table S41). NBS-type proteins in Ppus were further divided into 27 CC-NBS (CN)-type, 29 CC-NBS-LRR (CNL)-type, 65 NBS-type, 95 NBS-LRR (NL)-type, 11 RPW8-NBS-LRR (RNL)-type, 13 RPW8-NBS (RN)-type, 53 TIR-NBS (TN)-type, 81 TIR-NBS-LRR (TNL)-type, 19 TIR-unknown domain/site (TX)-type, and 11 ‘other types’ (Fig. 5B; Supplementary Data Table S42). Over half of the TN-, TNL-, and CNL-type transcripts were on chromosomes (Chrs) 2 and 8; two-thirds of TN-type transcripts were on Chrs 1, 2, and 8; and one-third of TNL-type transcripts were on Chr 2 (Supplementary Data Table S42).

We also detected 1397 RGAs in Pavi, namely 277 NBS-type, 715 RLK-type, 229 RLP-type, 17 RPW8-type, and 159 TM-CC-type proteins (Fig. 5C; Supplementary Data Tables S43 and S44). Hence, Pavi had more RGAs than Ppus (*n* = 1330). However, Ppus had more NBS-type RGAs (*n* = 404) than Pavi (*n* = 277). We performed a more detailed analysis of NBS-type proteins in Pavi and identified 13 CN-type, 82 CNL-type, 43 NBS-type, 78 NL-type, 15 RNL-type, 4 RN-type, 5 TN-type, 17 TNL-type, 13 TX-type, and 7 other types (Fig. 5D; Supplementary Data Table S45). Of these, fewer TNL-type (*n* = 17), TN-type (*n* = 5), and TX-type (*n* = 13) proteins were found in Pavi than Ppus (Supplementary Data Tables S42 and S45), suggesting that the two types of RGAs in Ppus has undergone a huge expansion during evolution. We analyzed the collinearity of TNL-type genes between the Ppus and Pavi genomes (Fig. 5E) and found 10 collinear TNL-type gene pairs on Chr 8 (Supplementary Data Fig. S6A; Supplementary Data Table S46). Furthermore, we also analyzed the NL-type genes between the Ppus and Pavi genomes (Fig. 5F) and found 17 collinear pairs on Chr 2 (Supplementary Data Fig. S6B; Supplementary Data Table S46). Some TNL-type proteins participate in the recognition of specific pathogens and play crucial roles in *P. syringae* resistance [23, 36]. Some TN- and TX-type proteins that participate in plant defenses might cooperate with TNL proteins to facilitate pathogen recognition or downstream signaling [37]. Thus, Ppus has evolved more TNL-, TN-, and TX-type transcripts than Pavi, which explains to some degree why Ppus is more resistant to *Pst* DC3000.

### WRKY family in *P. pusilliflora*, *P. persica*, *P. serrulata*, and *P. yedoensis*

The WRKY family of proteins, first discovered in plants, is characterized by a sequence of 60 amino acids that includes the WRKY domain [38]. These proteins play vital roles in pathogen defense and the environmental stress response, and development [15, 16, 39]. Genome-wide analysis of WRKY has been performed in several plants, including maize [40], peaches [41], *Camellia sinensis* [42], and strawberry [17]. We identified 61 WRKY genes in the Ppus genome, 58 in Pper, 60 in Pser, and 78 in Pyed (Supplementary Data Table S47). We then categorized them based on phylogeny and structural domains into group I WRKY, consisting of subgroups Ia and Ib, group II consisting of subgroups IIa, IIb, IIc, IId, and IIe, and group III (Supplementary Data Fig. S7A). In Ppus, subgroup Ia contained 3 genes; subgroup Ib, 7; subgroup IIa, 3; subgroup IIb, 8; subgroup IIc, 13; subgroup IId, 8; subgroup IIe, 8; and group III, 11 (Supplementary Data Table S47). We then compared 11 species to further investigate the evolution and divergence of WRKY. Most WRKY subgroups were found in all species, although *Oryza sativa* did not have subgroup IIe. Expansion of the WRKY groups was observed in groups I and III and subgroup IIc (Supplementary Data Fig. S7B). Notably, *Giardia lamblia* and *Dictyostelium discoideum* each only contain one known *WRKY* gene, whereas gene duplication in *Physcomitrella patens* has resulted in an increase of 37 WRKY proteins [40]. The WRKY genes rapidly duplicated before monocots and dicots diverged [43]. Monocots also have larger WRKY families than most dicotyledons (Supplementary Data Fig. S7B). For instance, maize contains the largest WRKY family of 136 genes, whereas Ppus has 61. The rapid duplication of WRKY genes, as vital TFs, might contribute to enhancing disease resistance, environmental stress adaptability and establishing a better stress-resistance signaling network.

### Cytochrome P450 family in *P. pusilliflora*, *P. persica*, *P. serrulata*, and *P. yedoensis*

The cytochrome P450 (CYP450) family catalyzes the biosynthesis of numerous important plant compounds, which are categorized into A- and non-A-types and further subdivided into clans [44, 45]. In *Arabidopsis*, A-type CYP450 contains clan 71, while the non-A-type consists of clans 51, 72, 74, 85, 86, 97, 710, and 711 [46]. We identified 263, 271, 288, and 432 *CYP450* genes in Ppus, Pper, Pser, and Pyed, respectively (Supplementary Data Table S48). Phylogenetic analysis indicates that Ppus CYP450s are also grouped into A-type (clan 71), consisting of 18 subfamilies, and non-A-type with clans 72, 711, 86, 97, 85, 710, 51, and 74, consisting of 24 subfamilies (Supplementary Data Fig. S8). We further explored CYP450 evolution and divergence between Ppus and 14 other species. The *CYP701*, *CYP84*, *CYP72*, *CYP714*, *CYP704*, and *CYP88* genes were most abundant in Ppus.

**Figure 6:**
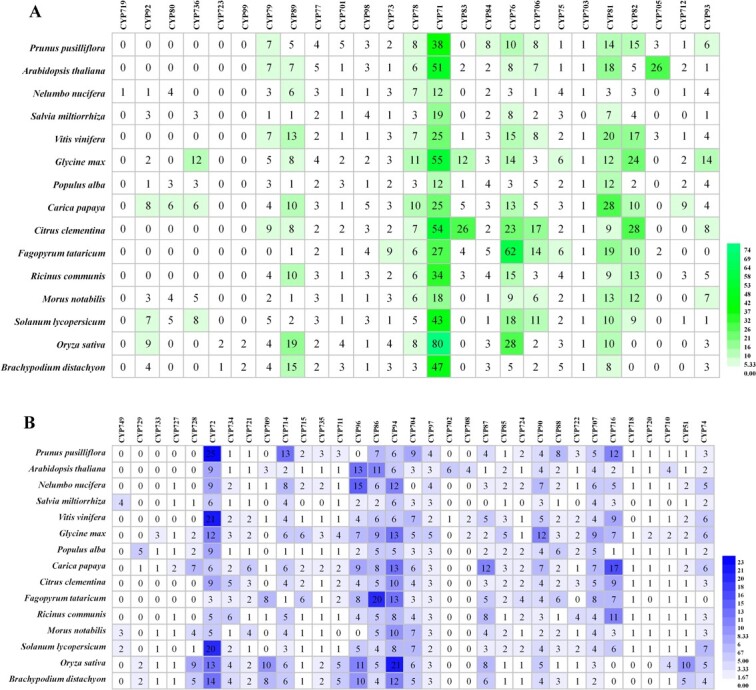
Distribution of CYP450 gene family sizes in the whole genome for 15 species: *P. pusilliflora*, *A. thaliana*, *Nelumbo nucifera*, *Salvia miltiorrhiza*, *V. vinifera*, *Glycine max*, *Populus alba*, *Carica papaya*, *Citrus clementina*, *Fagopyrum tataricum*, *Ricinus communis*, *Morus notabilis*, *Solanum lycopersicum*, *Oryza sativa*, and *Brachypodium distachyon*. (**A**) A-type CYP450. (**B**) Non-A type CYP450.

The CYP99 and CYP723 subfamilies were found only in monocots, whereas CYP82 and CYP716 were found only in dicots (Fig. 6). Twenty-four CYP subfamilies (e.g. CYP89, CYP77, CYP71, CYP81, and CYP76) were common to all 15 species. CYP719 was found only in *Nelumbo nucifera*. These results showed that some CYP subfamilies (i.e. CYP79, CYP93, and CYP74) were lost only in a single species. The CYP71, CYP72, CYP76, CYP81, and CYP94 subfamilies expanded massively (Fig. 6). Notably, the CYP71 family converts aldoximes to nitriles that participate in resistance to biotic stress [47]. Thus, the rapid duplication of some CYP subfamilies might contribute to improved stress tolerance in plants.

### MADS-box family in *P. pusilliflora*, *P. persica*, *P. serrulata*, and *P. yedoensis*

MADS-box family genes are vital to plant development, especially during dormancy release and the development of flowers and fruits [20, 48]. In plants, MADS-box genes are divided into types I and II lineages based on protein domain structures [49]. Type II MADS-box genes have a conserved MADS-box domain, intervening (I) and keratin-like (K) domains, and a C-terminal (C) region that are sequentially arranged from the N- to the C-termini; these genes are also called MIKC-type genes [48, 50]. These genes are further subdivided into MIKC^C^ and MIKC* types. In *Arabidopsis*, MIKC^C^-type genes are categorized into 12 subfamilies [51, 52]. Type I MADS-box genes are classified into Mα, Mβ, Mγ, and Mδ groups based on phylogeny. The Mδ group in *Arabidopsis* and rice corresponds to the MIKC* type [53].

**Figure 7:**
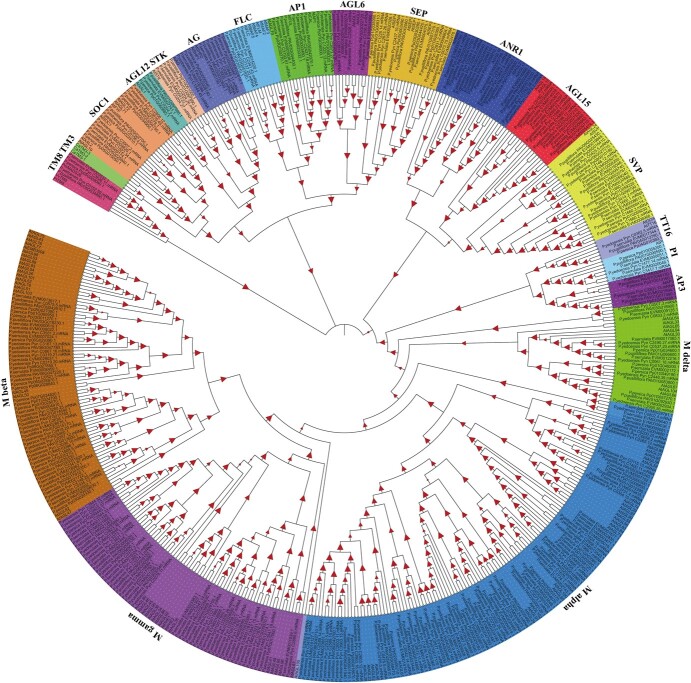
A phylogenetic tree of MADS-box gene family members in *P. pusilliflora*, *P. serrulata*, *P. yedoensis*, and *A. thaliana*. Grapevine TM8 (TC62855), poplar TM8 (XP_002321711.1), and *P. mume* PmMADS26 (Pm024524) were used for this phylogenetic analysis because the *Arabidopsis* genome lacks the TM8 subfamily. *Coffea arabica* TOMATO MADS-box 3 (TM3), CaTM3-1 (KJ483226), CaTM3-2 (KJ483227), and CaTM3-3 (KJ483228) were also used to construct the phylogenetic tree.

MADS-box family genes are reported in multiple *Prunus* species [49, 54] but remain undescribed in Ppus and Pyed. Herein, we detected 81 MADS-box members in the Ppus genome, 77 in Pper, 97 in Pser, and 131 in Pyed (Supplementary Data Table S49). In accordance with the Atha classification, we divided type I MADS-box genes into Mα (18), M-β (11), M-γ (11), and M-δ (4) groups in Ppus (Fig. 7). We also categorized Ppus type II MADS-box members into 15 subfamilies based on phylogeny: *ARABIDOPSIS NITRATE REGULATED 1* (*ANR1*), *SHORT VEGETATIVE PHASE* (*SVP*), *AGAMOUS-like 15* (*AGL15*), *SUPPRESSOR OF OVEREXPRESSION OF CONSTANS 1* (*SOC1*), *AGL12*, *SEEDSTICK* (*STK*), *AGAMOUS* (*AG*), *FLOWERING LOCUS C* (*FLC*), *APETALA1* (*AP1*), *AGL6*, *SEPALLATA* (*SEP*), *TRANSPARENT TESTA 16* (*TT16*)/*AGL32*, *APETALA3* (*AP3*), *TOMATO MADS-box 8* (*TM8*), and *PISTILLATA* (*PI*) (Fig. 7). Among these subfamilies, 14 were grouped with their *Arabidopsis* counterparts. We used grapevine *TM8*, poplar *TM8* (XP_002321711.1), *P. mume PmMADS26* (a homologous gene of *TM8*), and *Coffea arabica TOMATO MADS-box 3* (*TM3*) for phylogenetic analysis because the *Arabidopsis* genome lacks the TM8 and TM3 subfamilies [49, 52, 55]. The MADS-box PAV05G034890 of Ppus unambiguously grouped with these three TM8 genes (Fig. 7), indicating that the Ppus genome has only one TM8 member, similar to *P. mume* and grapevine. The Ppus, *Arabidopsis*, Pper, Pyed, and Pser genomes notably have no genes that are homologous to TM3, suggesting that this subfamily might be unique to *C. arabica*. The most expanded type II MADS-box subfamilies were SVP and AGL15, with four each in Ppus and two in Atha. Considering that SVP is important for early flowering during spring, we speculated that the expansion of this subfamily in Ppus is correlated with the control of flowering time. Evolutionary analysis showed that four Ppus MADS-box TFs (i.e. PAV06G025430, PAV08G027210, PAV01G095740, and PAV01G095790) clustered with AtSVP in a clade, suggesting that these four proteins play important roles in the regulation of bud endodormancy.

## Discussion

We successfully addressed a gap in knowledge about wild cherry genomes and generated a high-quality chromosomal-level Ppus genome using NGS, ONT, and Hi-C sequencing technologies [56, 57]. Because of the strong disease resistance of this species, it is an important germplasm resource for cherry breeding programs, and decoding its genome sequence is of great significance. Due to data limitations, we did not generate a haplotype-resolved assembly in this study, but assembly quality is comparable to or even better than that of other published cherry species [27] (Supplementary Data Table S50). We found that the assembled genome of Ppus (309.62 Mb) was smaller than those of Pyed [27] (323.8 Mb) and Pavi [3] (344.29 Mb) but larger than that of Pser [26] (265.4 Mb). The Ppus genome had a higher repetition rate (49.08%; 309.62 Mb) than the genomes of *Prunus* species Pdul [29] (34.6%; 227.6 Mb), *P. mume* [32] (45.0%; 280 Mb), and Pper [58] (37.14%; 265 Mb), which might help explain why Ppus has a larger genome than these species. Additionally, Ppus has higher scaffold N50 (33.87 Mb) and contig N50 (6.81 Mb) values than Pyed [27] (scaffold N50 = 198.95 kb, contig N50 = 132.59 kb), Pser [26] (scaffold N50 = 31.12 Mb, contig N50 = 1.56 Mb), and Pavi [3] (scaffold N50 = 42.62 Mb; contig N50 = 3.25 Mb). Our Ppus assembly will serve as a high-quality reference genome for further investigations regarding cherries. Moreover, this genome assembly provides necessary data for clarifying the genetic background and evolution of Ppus, Pyed, Pser, and Pavi and the independent domestication of cherries.

Phylogenetic analysis with high-quality single-copy orthologs revealed that the *Cerasus* species Ppus, Pavi, Pser, and Pyed clustered on a branch with the shortest divergence time and were separate from the *Prunus* species Pper, Pdul, and Parm. Both the phylogenetic tree and *K*_s_ analysis indicated that Pavi diverged earlier than Pser and Pyed, which was consistent with previous findings [26]. Our results showed that Ppus was more closely related to Pser than to Pavi, which is supported by a perfect collinear relationship between Ppus and Pser. We also found that all syntenic blocks between Ppus and Pser matched on the same chromosome, compared with only 65.77% of those between Ppus and Pavi (Supplementary Data Table S19). We did not find any large-scale chromosome inversions or translocations between the Ppus and Pser genomes but did for the Ppus and Pavi genomes (Fig. 2). One potential reason is that Pavi underwent more artificial selection than Pser, resulting in inversions and other chromosome structure variations, though further research is needed to confirm these inversions. The relationship between Ppus and Pper is farther than that between Ppus and Pavi, but Ppus had good collinearity with Pper, possibly due to having fewer gene syntenic blocks with which to build the collinear relationship between Ppus and Pper.

Disease resistance strongly depends on R genes in plants [13]. We found that wild Ppus was more resistant to *C. viniferum*, *P. capsici*, and *Pst* DC3000 than was cultivated Pavi. Although the number of RGAs was comparable between Ppus and Pavi, the former species had considerably more NBS-type RGAs. The majority of the NBS type confer resistance to pathogenic viruses, bacteria, oomycetes, and fungi [14]. Thus, we speculated that NBS-type expansion partially enhanced Ppus resistance to some pathogens.

The TNL-type proteins RPS4 and RPS6 recognize *P. syringae* effectors and confer resistance to *P. syringae* [36]. The TN- and TX-type proteins cooperate with TNL proteins to facilitate pathogen recognition or downstream signaling [37]. Here, Ppus contained more TNL-, TN- and TX-type transcripts than did Pavi, which indicated that the expansion of TNL, TN and TX types might confer on Ppus stronger resistance to *Pst DC3000*. Some RLP-type RGAs, such as RLP30 and RLP42, are essential for resistance to *B. cinerea* [59, 60]. We found that Ppus had fewer RLP-type proteins than Pavi, which explains why Ppus is somewhat less resistant to *B. cinerea*. Some RLKs play important roles in plant resistance to pathogens [61]. In fact, RLK1 is involved in the hypersensitivity response signaling pathway and functions in *P. capsici* resistance [62]. We found that Ppus had fewer RLK-type transcripts than Pavi but was more resistant to *P. capsici*, implying that more dominant genes are involved in resistance to *P. capsici* in Ppus. Further identification of RGAs will enable us to determine the resistance traits of various types of R genes and apply these findings to breeding programs.

AtWRKY3 and AtWRKY4 are positive regulators of resistance against the necrotrophic fungus *B. cinerea* [63, 64]. Evolutionary analysis showed that the Ppus WRKY protein PAV01G067110 was closely associated with AtWRKY3 and AtWRKY4, implying it functioned in *B. cinerea* resistance. AtWRKY38, AtWRKY48, and AtWRKY62 negatively influence basal resistance toward virulent *P. syringae* [65, 66]. Our results showed that the Ppus WRKY protein PAV02G008190 was closely related to AtWRKY38 and AtWRKY62 and that PAV01G017570 was closely associated with AtWRKY48, suggesting that they have similar functions in *P. syringae* defense.

AtCYP76C2 is associated with hypersensitive rapid cell death, which is a defense mechanism for *Pst* DC3000 infection [67]. Evolutionary analysis showed that several Ppus CYP450s (such as PAV01G058410 and PAV01G058390) were closely associated with AtCYP76C2, implying that they function in resistance to *Pst DC3000*. A pathogen-induced *CYP82C2* gene and other possible CYPs are involved in the biosynthesis of 4-hydroxyindole-3-carbonyl nitrile with cyanogenic functionality against *P. syringae* [68]. In soybean, *GmCYP82A3* is highly resistant to *B. cinerea* [69]. Our results revealed that 15 Ppus *CYP450*s and five *AtCYP82* genes clustered together, suggesting that they play important roles in resistance to *B. cinerea* and *P. syringae*.

As Ppus is an important ornamental tree species that grows during early spring, we focused on the MADS-box family in this study because of its involvement in dormancy release and floral organ development. We identified 81, 77, 97, and 131 MADS-box genes in Ppus, Pper, Pser, and Pyed, respectively. These gene numbers indicate that flowering cherry Pyed has well-developed floral organs, whereas Pper does not, probably because some MADS-box genes had been deleted over a long period of artificial selection. Loss of the TM3 subfamily might affect the transition from vegetative to reproductive growth in the four *Prunus* species Ppus, Pper, Pser, and Pyed. Because the SVP subfamily is associated with early flowering, its expansion suggests a need for better control of flowering time during the evolution of Ppus. Finally, DAM has been verified to have functions related to the inhibition of bud break in pears [20]; *DAM* genes, usually named *SVP* or *SVP*-like (*SVL*), mainly participate in the regulation of endodormancy [21]. Our results revealed that four Ppus MADS-box genes were closely related to *AtSVP*, implying that they function in regulating Ppus bud endodormancy.

## Materials and methods

### Plant materials and DNA extraction

We used DNeasy Plant Mini Kits (Tiangen Biotech Co. Ltd, Beijing, China) to extract high-purity genomic DNA from the fresh and young leaves of an endemic wild Ppus tree aged ~120 years preserved in its natural habitat (Binchuan County, Dali District, Yunnan Province, China). The concentration and purity of extracted DNA were evaluated using a Nanodrop 2000 spectrophotometer (Thermo Fisher Scientific, Waltham, MA, USA) and a Qubit 3.0 fluorometer (Thermo Fisher Scientific, Waltham, MA, USA). DNA completeness was assessed by 0.8% agarose gel electrophoresis using pulsed-field techniques.

### Genomic DNA sequencing

We constructed a paired-end library using GenElute Plant Genomic DNA Miniprep Kits (Sigma–Aldrich, St Louis, MO, USA) for short-read sequencing based on the Illumina HiSeq X Ten (Illumina, San Diego, CA, USA) platform. On the other hand, an ONT library was constructed for long-read sequencing using an Oxford Nanopore PromethION 48 platform (Oxford Nanopore Technologies, Oxford, UK) at Novogene Co., Ltd (Beijing, China). A Hi-C library was generated as follows. First, fresh and young leaves fixed in formaldehyde were lysed, then cross-linked DNA was digested overnight using the single four-cutter restriction enzyme *Dpn* II. Then, digested fragments were ligated and biotinylated to form chimeric rings, which were enriched, sheared, and further processed. The Hi-C library was also sequenced based on the Illumina HiSeq X Ten platform. Raw reads were subjected to quality control procedures that involved adapter trimming and removal of low-quality reads. The resultant clean reads were used for subsequent analysis.

### *De novo* genome assembly

We estimated the genome size and repeat ratio using Jellyfish v2.2.10 [70] and GenomeScope v2.0 [71]. Oxford Nanopore long-read data were assembled into a genome *de novo* using NECAT v0.0.1 (https://github.com/xiaochuanle/NECAT) with default parameters [72]. The workflow for NECAT involved raw read correction, contig assembly, and bridge contigs. The resultant genome assembly was polished using Racon3 with clean short reads as the input [57]. Two rounds of polishing and purging were performed by using purge_dups v1.2.5 (https://github.com/dfguan/purge_dups) to produce a non-scaffolded draft assembly. We performed Hi-C chromosome conformation capture using Juicer v1.6.2 and 3D-DNA v180922 with default parameters, using ~87.9 Gb data to enhance assembly contiguity [73]. We then assessed the reliability of the Hi-C-based chromosomal assembly by utilizing Juicebox’s chromatin contact matrix v2.18.00 (https://github.com/aidenlab/Juicebox) [74].

### Genome assembly evaluation

We used the Burrows–Wheeler Aligner with default parameters [75] to align Illumina reads to the assembly for estimating the coverage ratio. Additionally, we evaluated the completeness and quality of the genome and annotated proteins using BUSCO v3.0.1 with default parameters by mapping them to the embryophyta_odb10 database [76].

### Repeat annotation

We predicted repetitive elements using *ab initio* and evidence-based methods. For the *ab initio* approach, we utilized LTR_FINDER v1.05 [77], LTRharvest v1.5.10, and LTR_retriever v1.8.0 [78] to identify the complete 5′- and 3′- ends of LTR elements. Meanwhile, the LAIs were calculated using LTR_retriever v1.8.0. We used RepeatModeler v2.0.10 [79] for the prediction of novel repeat elements. To predict repetitive sequences in the genome, we employed evidence-based approaches using RepeatMasker v4.0.9 and RepeatProteinMask v4.0.9 (http://www.repeatmasker.org). These programs utilized a repeat library downloaded from the database of Repbase v21.12 (https://www.girinst.org/downloads/) [80]. We identified and annotated tandem repeats by using the tool of Tandem Repeats Finder (TRF, v4.09) [81]. RepeatMasker v4.0.7 was utilized to predict *ab initio* repetitive elements using a *de novo* repeat library combining the results of LTR_retriever v1.8.0 and RepeatModeler v2.0.10. The chromosomal distribution of repeats was calculated using a sliding window of 1 Mb.

### Gene annotation and functional annotation

We predicted protein-coding genes of the Ppus genome using homology-based, *de novo*, and transcriptome-based methods. Gene models were predicted using GeneWise v2.4.1 [82] based on the homology of Pavi proteins downloaded from NCBI [83]. We then predicted transcriptome-based gene models using StringTie v1.3.4 based on the homologous transcriptomes from NCBI [84]. Pavi PRJNA595502, PRJNA419491, PRJNA550274, and PRJNA73727, *Prunus cerasus* PRJNA327561 and PRJNA295439, and *Prunus subhirtella* PRJNA596558 were mapped to the genome using the software of HISAT v2.0.4 [85]. We utilized several tools, including GeneID v1.4.4 [86], GenScan v1.0 [87], GlimmerHMM v3.0.3 [88], Augustus v2.5.5 [89], and SNAP v1.0 [90] for *de novo* prediction. We integrated the results of the three methods using EvidenceModeler v1.1.1 [91]. Based on domain conservation and sequence similarity, we predicted gene model functions using the BLAST tool against the KEGG, UniProt, KOG, PFAM, TrEMBL, EggNOG, NR, and TAIR databases. To identify the gene ontology and domains of the gene models, we used InterProScan and data from multiple sources, including PRINTS, Pfam, PROSITE, SMART, GO, and ProDom [92]. We also predicted non-coding RNAs in the Ppus genome. snRNA and miRNA were predicted by using INFERNAL v1.1.2 [93] and covariance models from Rfam [94]. Transfer RNA (tRNA) predictions were conducted using tRNAscan-SE v2.0.2 [95]. Ribosomal RNAs (rRNAs) were predicted via a homology search using BLAST+ 2.2.29 with an E-value cutoff of 1e−10.

### Synteny analysis

To investigate genome collinearity, we compared the Ppus genome with those of Pper, Pavi, and Pser using MUMmer v4.0.0 (http://mummer.sourceforge.net) with the parameters -i 89 -l 1000. We visualized the results of genome collinearity using MUMmerplot v3.5. In addition, we analyzed gene synteny between the eight chromosomes of Ppus, Pper, Pser, and Pavi and generated gene syntenic blocks by comparing the Ppus genome with Pser, Pavi, and Pper genomes using the MCScanX software (https://github.com/tanghaibao/jcvi/wiki/MCscan-Python-version) with default parameters.

### Phylogenetic and gene family analysis

The longest protein sequences of Ppus and the nine other angiosperms, Pser [26], Pavi, Pyed, Parm (https://www.rosaceae.org), Pper [97], Pdul [29], Rchi [98], Vvin [99], and Atha [100], were clustered into orthologous groups using BLASTP (E-value = 1e−5) and OrthoMCL v2.0.9 [96]. To perform phylogenetic analysis, we aligned single copies of orthologous genes from the 10 species using MUSCLE v3.8.3 [101] with default parameters. A phylogenetic tree was then constructed using PhyML v3.0 with default parameters. To estimate the divergence time, we utilized the MCMCtree program from the PAML v4.9j package, with calibration based on the known divergence time from TimeTree (http://www.timetree.org) [102].

Gene families that had contracted or expanded were identified based on family size and phylogeny using CAFE v2.1 (parameters: number of threads = 10, *P* = 0.05, number of random = 1000, and search for lambda) [103]. Each gene module was subjected to functional enrichment analysis with GO and KEGG.

Seed files corresponding to the CYP450 (PF00067), WRKY (PF03106), and MADS-box (PF00319) gene families were obtained at the website of the Pfam database (http://pfam.janelia.org/). The domain file was used as the first template for scanning the gene families, and any output genes with an E-value of less than 1e−10 were filtered out. The filtered genes were taken as templates for a second scan, and then output genes were filtered out in the same way. Putative genes were identified in each gene family. The resulting sequences were aligned using MUSCLE v3.8.3 and the phylogenetic trees of gene families were constructed using FastTree v2.1.11 (http://www.microbesonline.org/fasttree/).

To identify R-genes in Ppus and Pavi, we used the RGAugury pipeline (https://bitbucket.org/yaanlpc/rgaugury) with default parameters to screen total filtered protein sequences from each species for the presence of RGAs [35]. We analyzed the NBS, RLP, RLK, RPW8, and TM-CC classes of RGAs. We identified the symmetry of functional resistance genes across the two species using MCscanX. We calculated R-gene density in a sliding window of 10 Mb using BEDTools v2.26.0 (https://bedtools.readthedocs.io/en/latest/index.html). The corresponding density graphs were visualized using the R package ggplot2 v3.2.1 (https://github.com/tidyverse/ggplot2).

### Polyploidization analysis

We adopted *K*_s_ to explore WGD and divergence events between *Prunus* and other species. Homologous amino acid sequences were aligned using MUSCLE v3.8.3 [101] and then converted into codon alignments using PAL2NAL v14 [104]. Finally, *K*_a_ and *K*_s_ were calculated via the Nei–Gojobori method using the NG86 program of PAML as described previously [105]. We used the median *K*_s_ between homologous genes to classify collinear blocks caused by duplication events. *K*_s_ was indicated via different colors on collinear blocks in WGDI [33]. Curves of *K*_s_ density distribution were created with Kspeaks (− kp). Multipeak fitting was conducted using the PeaksFit (− pf) software. Multiple fitted density curves were converted into one graph using KsFigures (− kf).

### Pathogen inoculation and disease development

*P. syringae* pv. *tomato* DC3000 was cultured on King’s B (KB) medium supplemented 50 μg/ml rifampicin at 30°C [106]. Log-phase cultures were resuspended with a buffer [10 mM MgCl_2_ and 10 mM 2-(*N*-morpholino) ethanesulfonic acid (MES)] to obtain an OD_600_ of 0.1 and then diluted 100-fold before spray inoculation. The sprayed leaves were monitored every 3 days for symptoms, and bacterial proliferation was measured in extracts of leaf tissues collected on 3 and 6 dpi. Three leaf disks with 5 mm diameter were collected from three independent leaves at 3 and 6 dpi and ground in 1 ml of 10 mM MgCl_2_ and 10 mM MES. Bacterial colonies were counted 2 days after plating 60 μl from serial dilutions on KB plates supplemented with rifampicin. To determine bacterial proliferation, we determined CFU/cm^2^ on each leaf at 3 and 6 dpi. In addition, the amount of *Pst* DC3000 DNA in plant DNA (%) was estimated using a previously described method [107, 108].

The *P. capsici* isolate LT263 was cultured on oatmeal agar at 25°C for 7 days. *C. viniferum* and *B. cinerea* were routinely cultured on potato dextrose agar at 25°C for 7–10 days. Agar disks (diameter 7.5 mm) were cut using a cork borer and then inoculated onto the abaxial surfaces of Ppus and Pavi cv. Tieton leaves that were maintained at 25°C. We collected the leaf disks with 5 mm diameter 5 mm away from agar disks and verified, using qPCR, if these pathogens had infected Ppus and Pavi [107, 108]. The primers used for qPCR are listed in Supplementary Data Table S51. Lesions were photographed at 3, 6, and 9 dpi, stained with lactophenol TB as previously described [109], and measured using ImageJ (National Institutes of Health, Bethesda, MD, USA).

### Statistical analysis

All data were statistically analyzed using SAS software (SAS Institute, Cary, NC, USA). We calculated statistical differences among all datasets by conducting a two-tailed Student’s *t* test, where *P* < .05 was used to denote significant differences. The results of the pathogen inoculation assays are shown as the mean ± standard deviation of values from more than nine replicates in each independent experiment.

## Acknowledgements

This study was funded by the Shanghai Agriculture Applied Technology Development Program, China (grant no. 2022-02-08-00-12-F01111), the China Agriculture Research System (grant no. CARS-30-2-08), the National Natural Science Foundation of China (grant no. 32102347), the Shanghai Sailing Program (grant no. 21YF1422100), and the Startup Fund for Young Faculty at SJTU (grant no. 21X010500643). We thank Dr. Xiaoyu Tu from Shanghai Jiao Tong University for proofreading the manuscript. We thank Dr. Wei Wu, Dr. Gang Yu, and Dr. Jiaqi Liu from Shanghai Jiao Tong University for their technical assistance. We thank Prof. Jiang Lu from Shanghai Jiao Tong University for providing the *C. viniferum*, *P. capsici*, *P. syringae* pv. *tomato* (*Pst*) DC3000, and *B. cinerea* samples used in this study.

## Authors contributions

C.Z. and Y.D. conceived and designed the experiments; S.J. collected the samples, performed disease resistance tests, and drafted the manuscript; B.C. and X.D. conducted the assembly and annotations and contributed to the sequencing data analyses; Z.L., Y.W., and Y.X. performed the disease resistance tests; C.Y., Z.Z., and S.L. collected the samples; M.L., J.Z., and J.W. worked on the phenotyping; S.Z., X.L., M.A.M., and W.S. performed the statistical analysis. R.L., L.W., G.Y., Q.J., E.D., Y.L., L.Y., and S.W. participated in discussions and provided valuable advice. All authors provided final approval for publication.

## Data availability

The raw genome sequencing data of Ppus are available at the National Genomics Data Center (https://ngdc.cncb.ac.cn/) under BioProject number PRJCA010538. All data are available from the corresponding authors upon request.

## Conflict of interest

The authors declare no competing financial interest.

## Supplementary data

Supplementary data is available at *Horticulture Research* online.

## Supplementary Material

Web_Material_uhad062
