# Supplementary material for: Chromosome-scale genome assembly of Prunus pusilliflora provides novel insights into genome evolution, disease resistance, and dormancy release in Cerasus L
Source: Hortic Res. 2023 Apr 10;10(5):uhad062. doi: 10.1093/hr/uhad062 (PMC10200261; doi:10.1093/hr/uhad062)
Supplement: Web_Material_uhad062 [file web_material_uhad062.zip › Supplementary Figures.pdf]

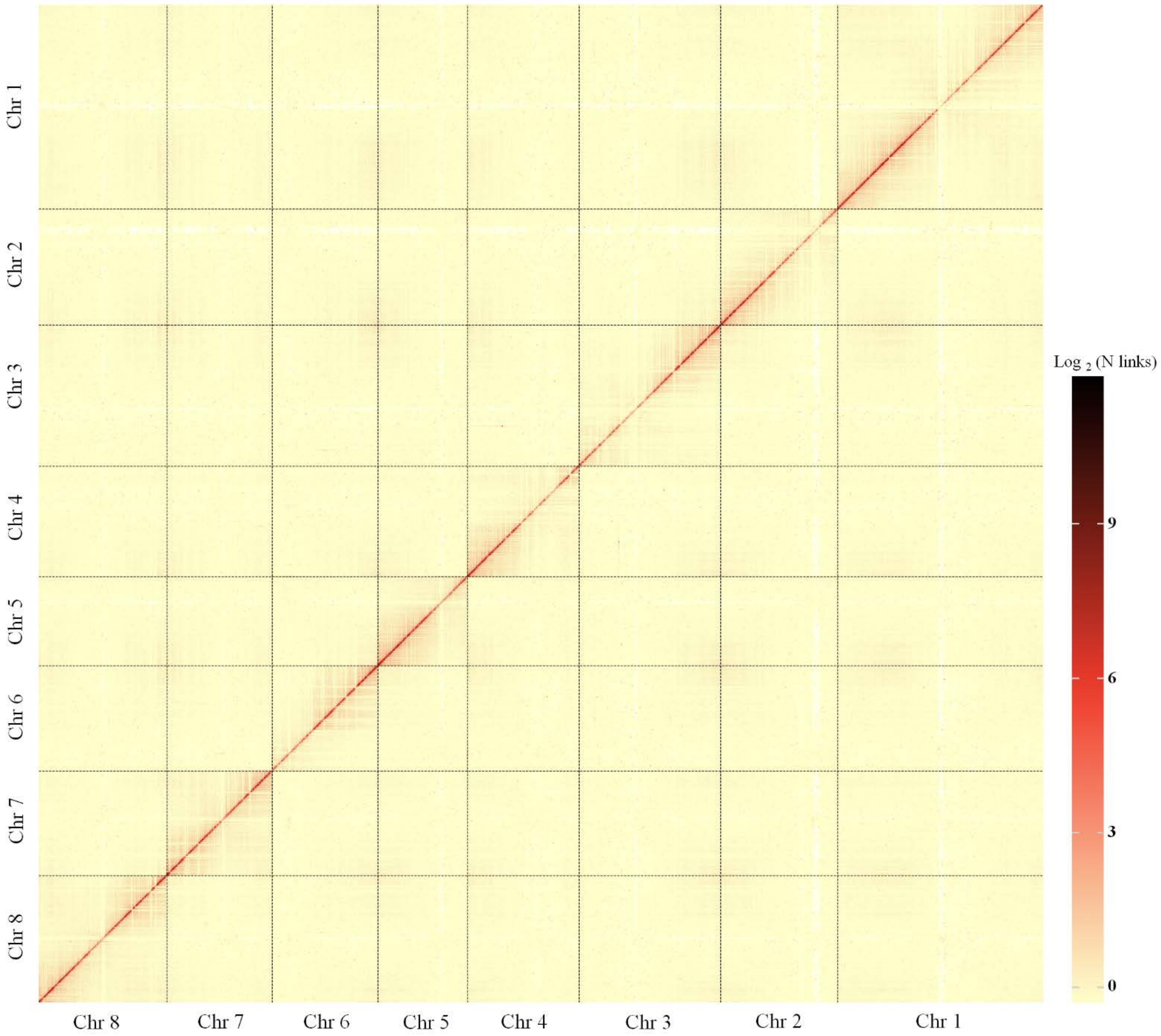

Fig. S1. High-resolution Hi-C interaction heatmap of *Prunus pusilliflora* genome. Individual chromosomes were scaffolded and assembled independently. Chr 1–8, chromosomes 1–8.

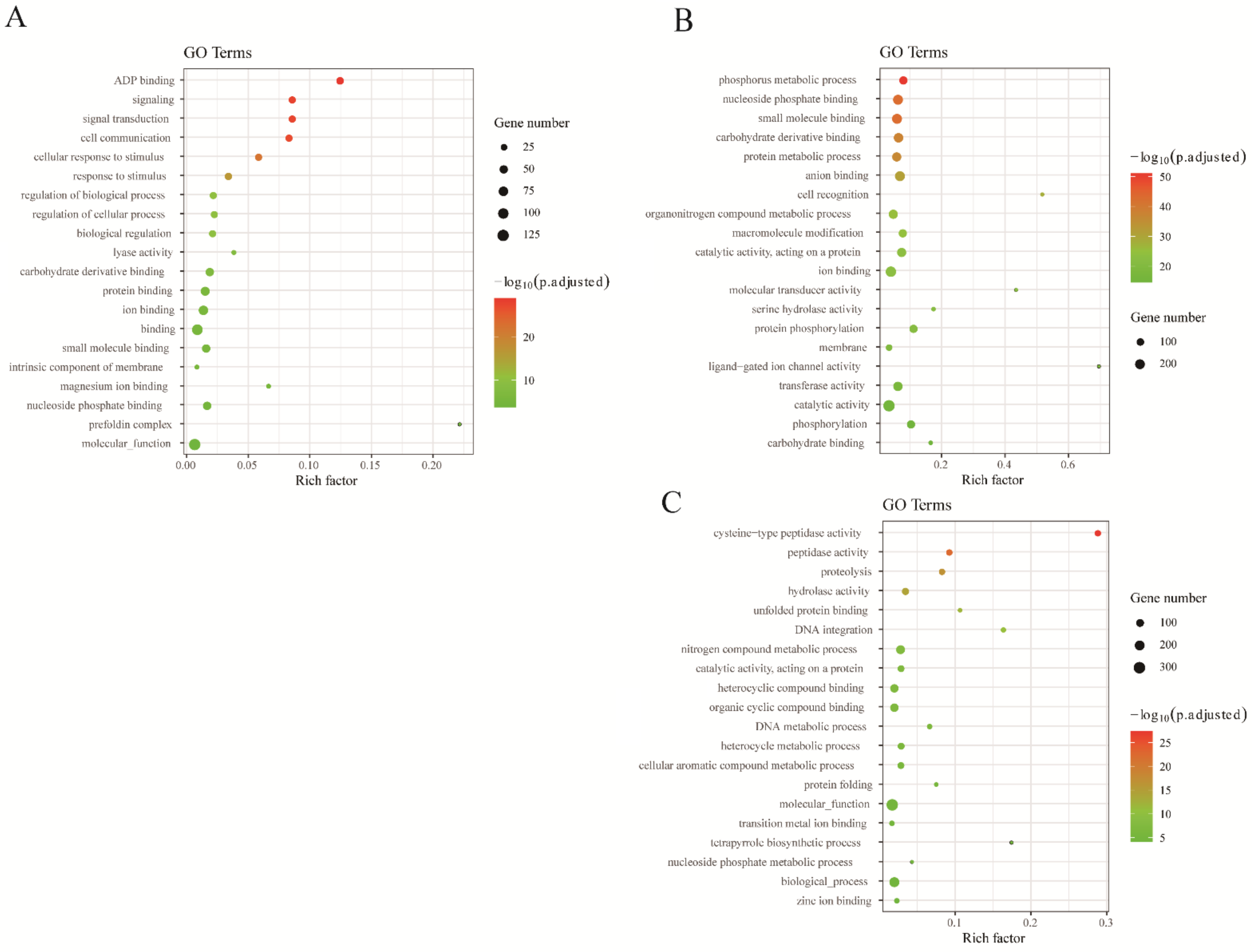

Fig. S2 Gene ontology (GO) enrichment analysis for the expanded (A), contracted (B), and unique (C) gene families in *Prunus pusilliflora*.

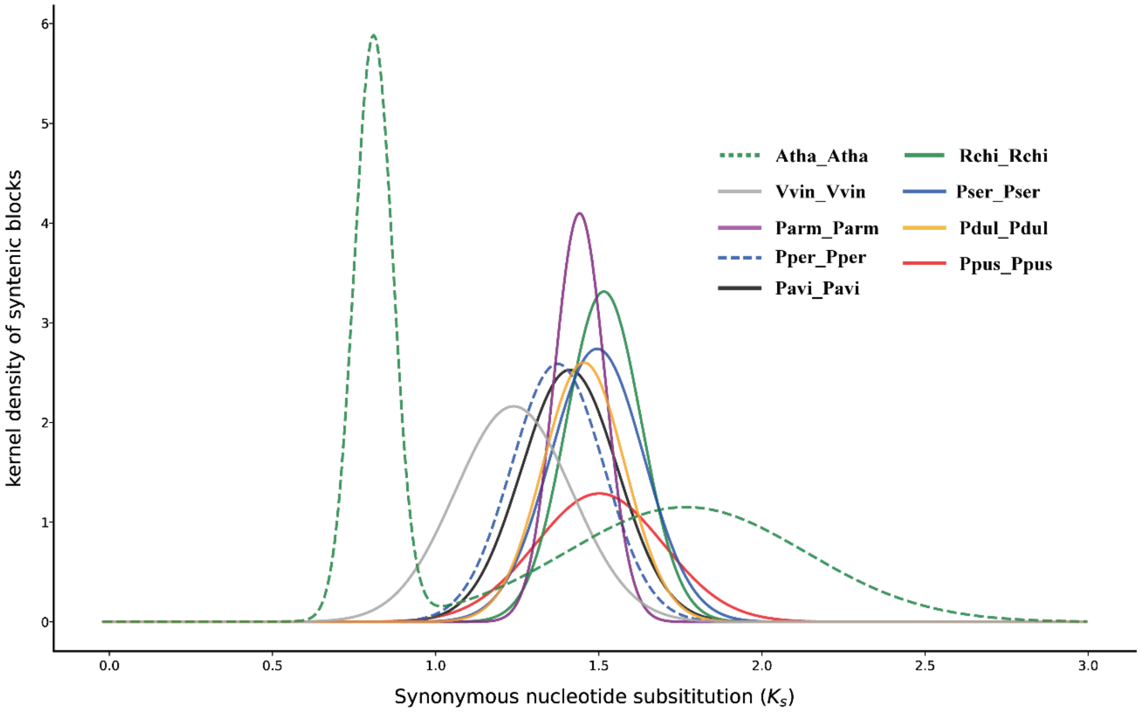

Fig. S3 The ks distribution of orthologs of *Prunus pusilliflora* and other species.

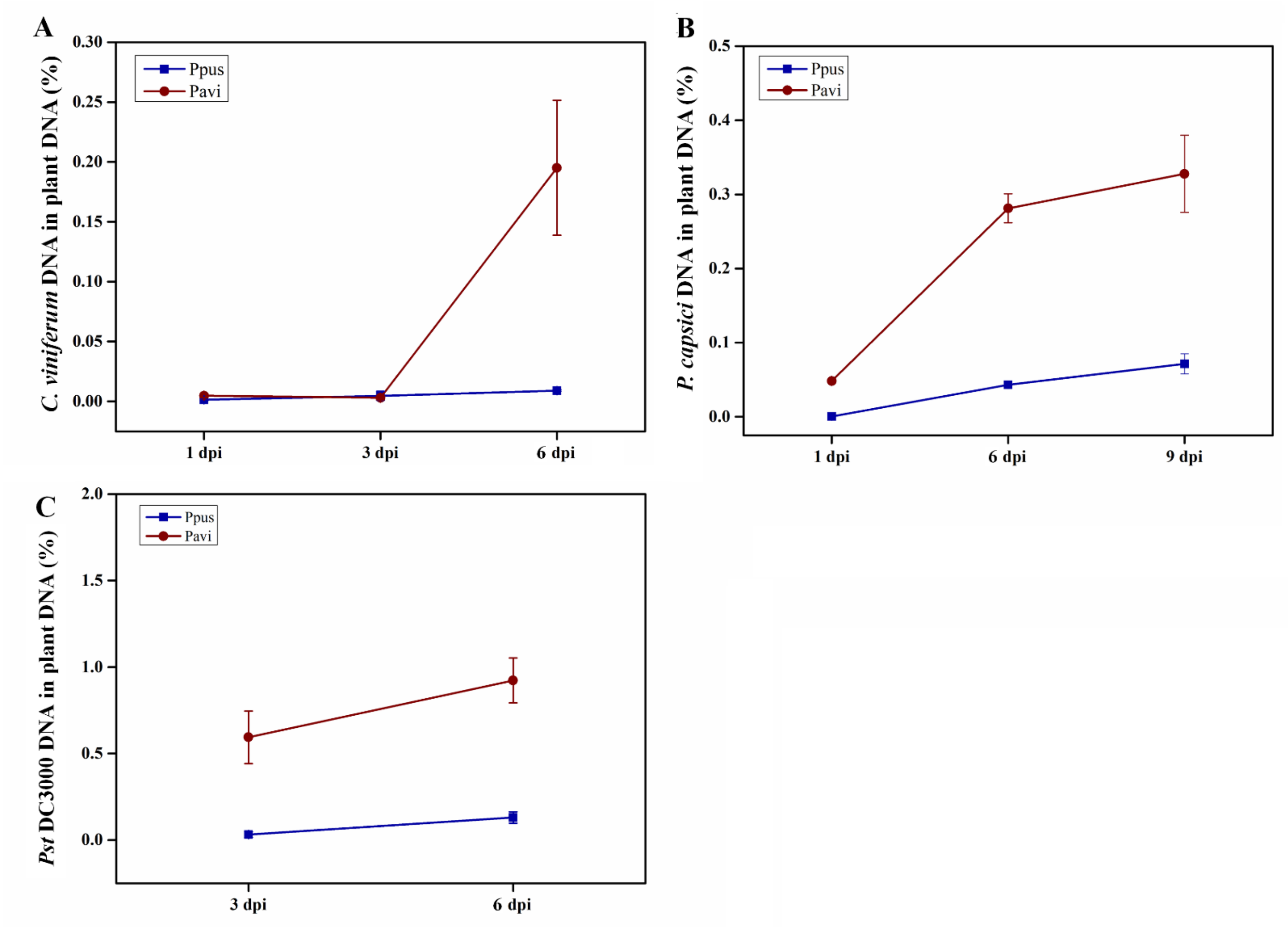

Fig. S4 Relative amounts of *Colletotrichum viniferum* (A), *Phytophthora capsici* (B), *Pseudomonas syringae pv. tomato DC3000* (C) DNA in *Prunus pusilliflora and P. avium* DNA. Data shown are the mean of four quantitative polymerase chain reaction (qPCR) replications ± standard deviation.

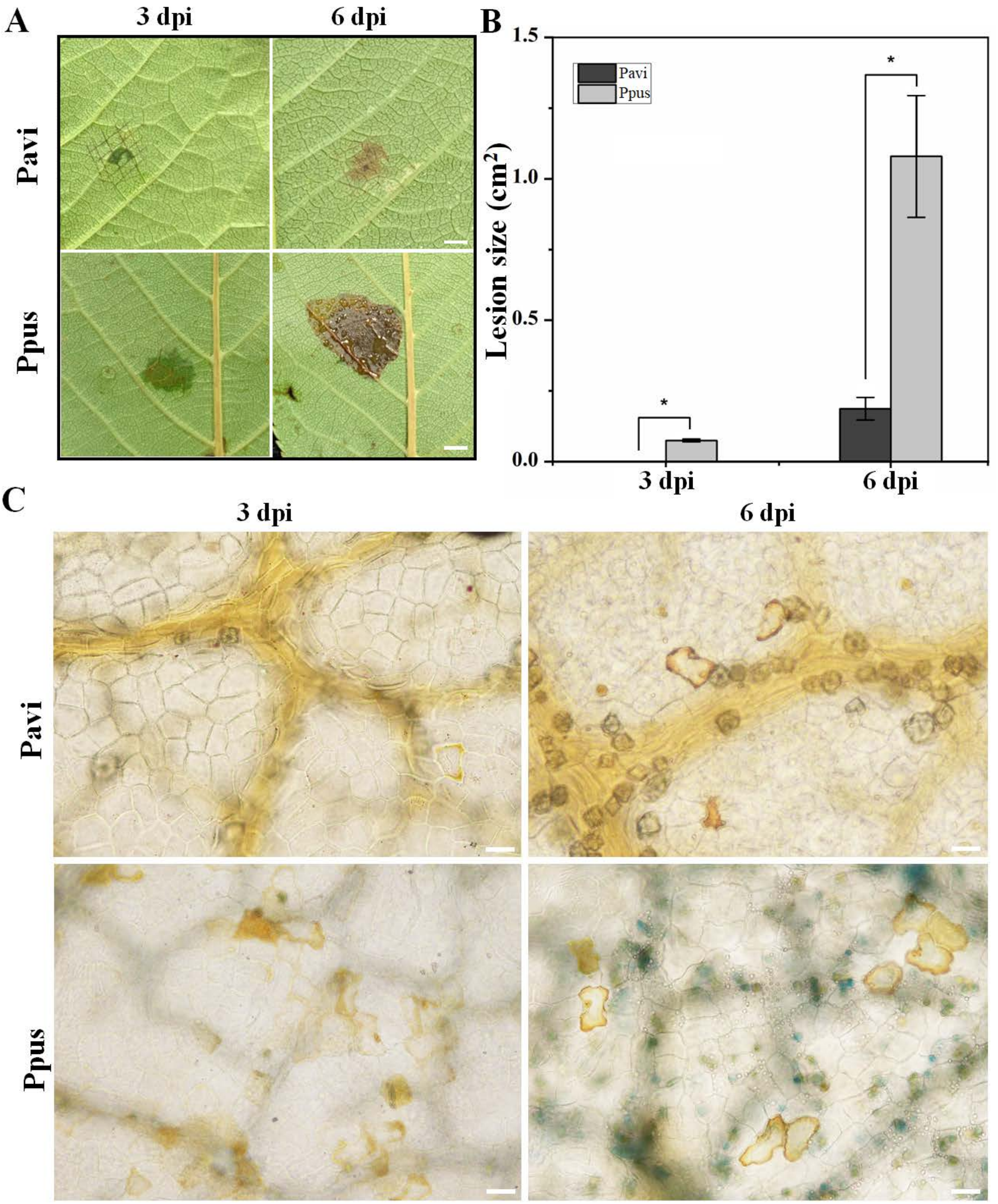

Fig. S5 Disease resistance evaluation of *Prunus pusilliflora* and *P. avium* leaves after inoculation with *Botrytis cinerea*. The lesions were photographed at 3, 6 days post inoculation (dpi) with *B. cinerea* and the lesion sizes were measured using Image J (National Institutes of Health, Bethesda, MD). Data represent mean ± standard deviation (SD) of each independent experiment with more than nine replicates. *$P < 0.05$ (two-tailed Student's *t* tests); ns, no significance. Scale bars, 0.5 cm. Pavi, *Prunus avium*; Ppus, *Prunus pusilliflora*. Leaves infected with *B. cinerea* were stained at 3 and 6 dpi with lactophenol Trypan Blue (TB) and then the area about 1 mm from lesions was photographed using a BX43 microscope (Olympus, Tokyo, Japan).

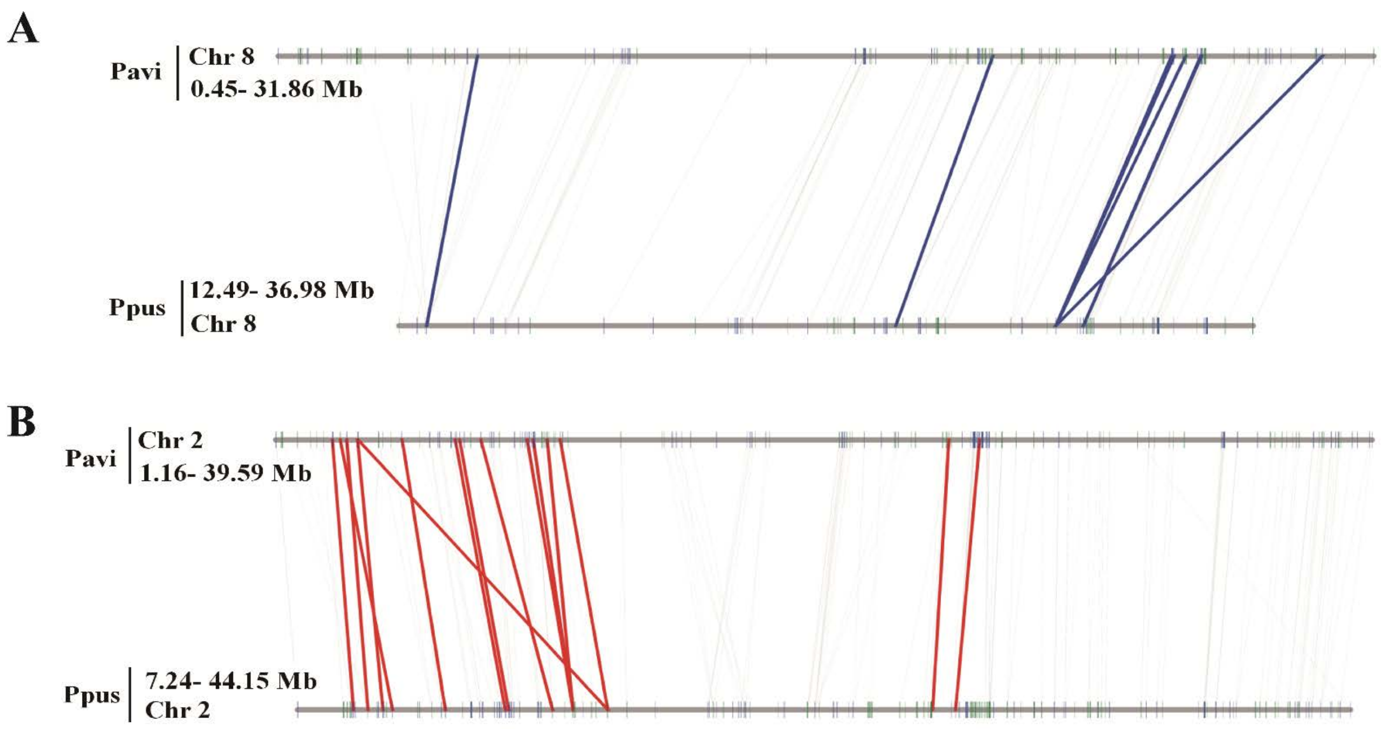

Fig. S6 Collinearity comparison of TNL-type genes on Chr 8 and NL-type genes on Chr 2 between the *Prunus pusilliflora* and *P*. *avium*.

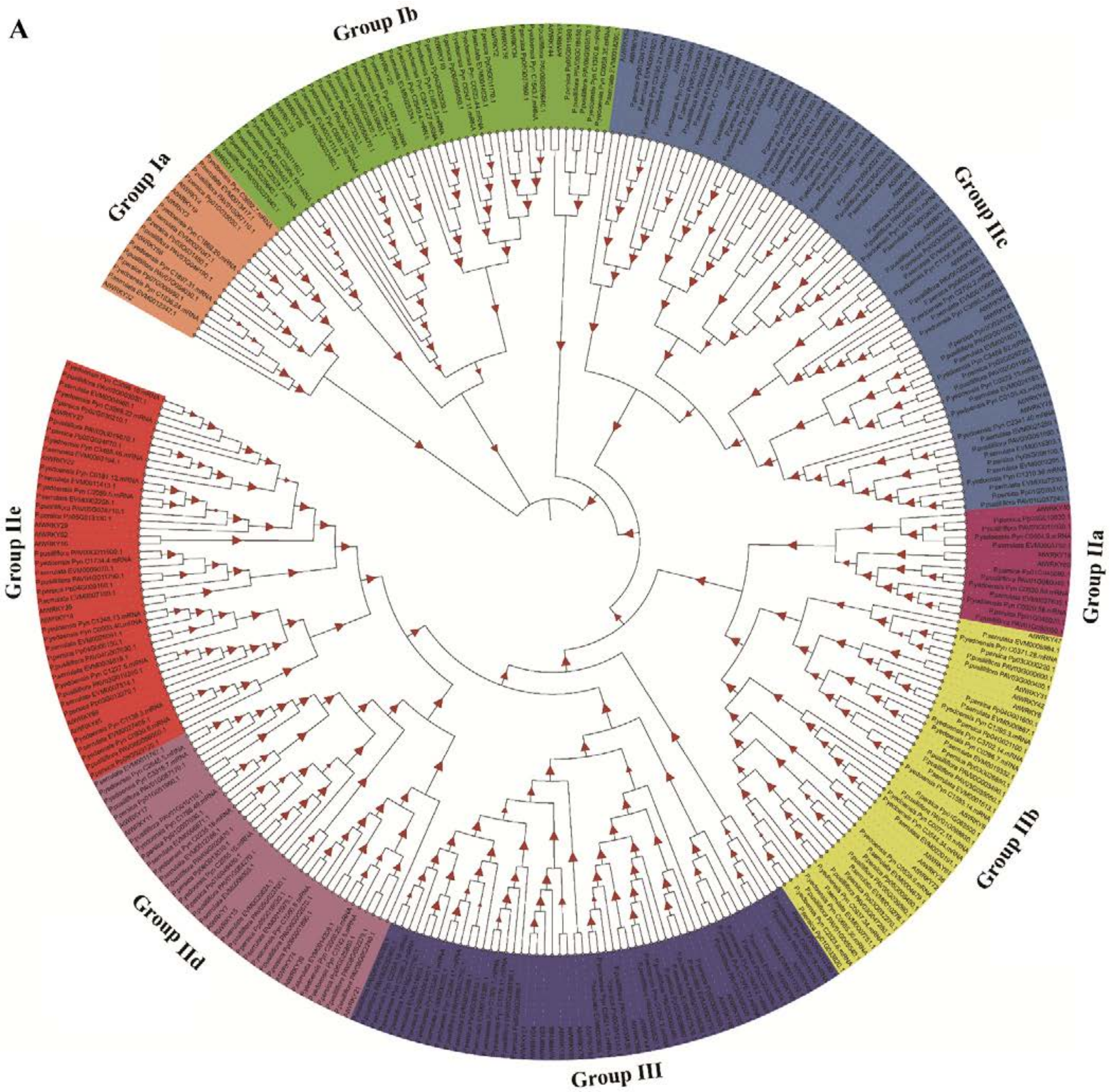

B

	PpuWRKY	*PpeWRKY*	*PsWRKY*	*PyWRKY*	*AtWRKY*	*AlWRKY*	*PtWRKY*	*CsWRKY*	*SlWRKY*	*OsWRKY*	*ZmWRKY*
I	10	12	9	17	16	13	50	10	15	34	22
II a	3	3	2	3	3	3	5	4	5	4	7
II b	8	8	8	11	8	8	9	4	8	8	11
II c	13	13	15	17	15	17	13	16	16	7	29
II d	8	7	7	8	7	7	13	8	6	11	14
II e	8	7	10	11	9	8	4	7	17	0	17
III	11	8	9	11	14	15	10	6	11	36	31

Color scale: 46, 43, 40, 36, 33, 30, 26, 23, 20, 16, 13, 10, 6.67, 3.33, 0.00

Fig. S7 WRKY family in *Prunus pusilliflora*, *P. serrulata*, *P. yedoensis*, and *Arabidopsis thaliana*. (A) Phylogenetic tree of WRKY in the four species. WRKY subfamilies are indicated with different colors. (B) The distribution of WRKY subfamily sizes in the whole genomes for 11 species: *P. pusilliflora* (*PpWRKY*), *P. persica* (*PpeWRKY*), *P. serrulata* (*PsWRKY*), *P. yedoensis* (*PyWRKY*), *A. thaliana* (*AtWRKY*), *A. lyrate* (*AlWRKY*) [110], *Populus trichocarpa* (*PtWRKY*) [111], *Cucumis sativus* (*CsWRKY*) [112], *Solanum lycopersicum* (*SlWRKY*) [113], *Oryza sativa* (*OsWRKY*) [43], and *Zea mays* (*ZmWRKY*) [40].

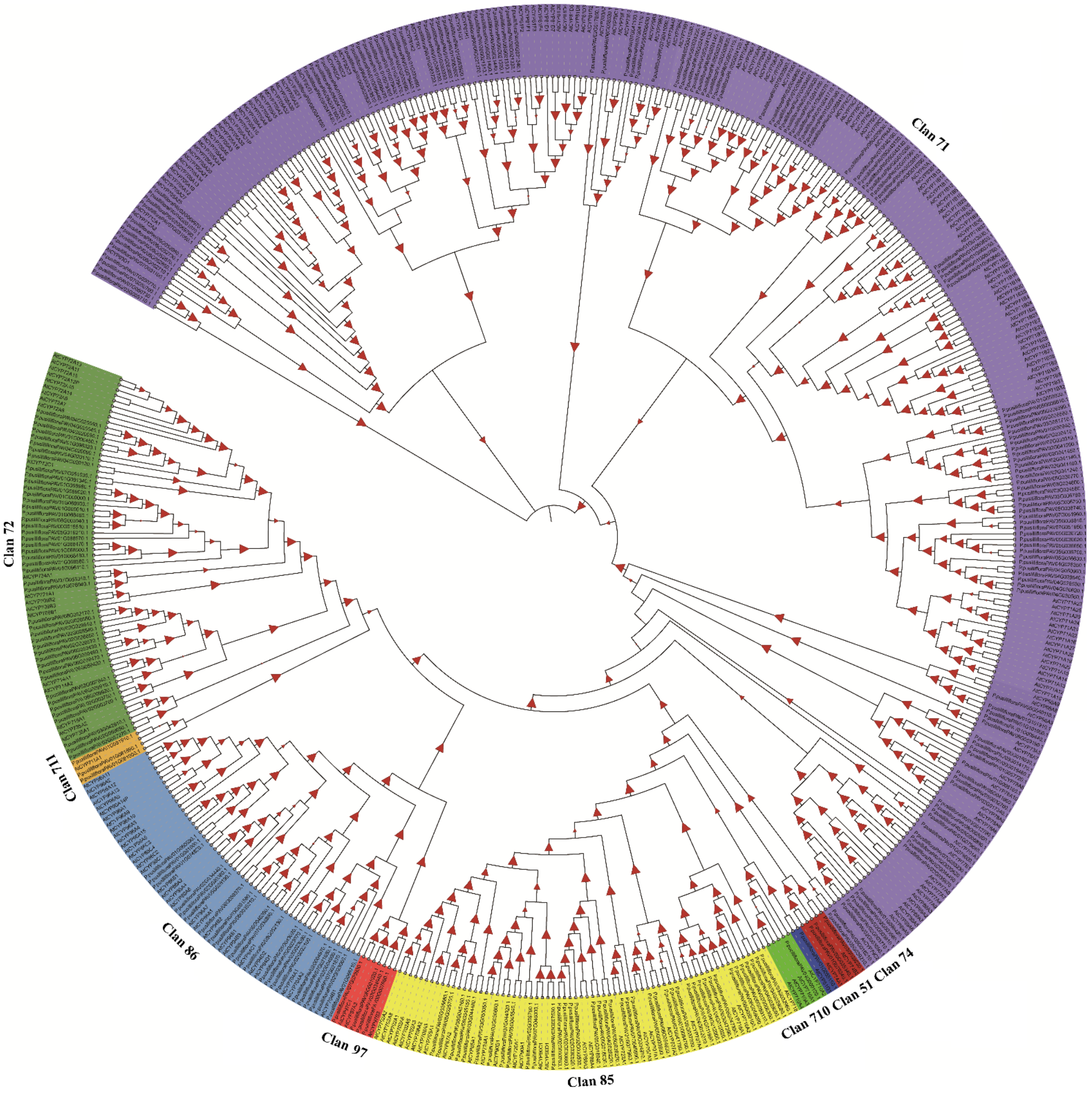

Fig. S8 Phylogenetic tree of the CYP450 family in *Prunus pusilliflora* and *Arabidopsis thaliana*. Each clan in the CYP450 family is shown with a different color.
