# Supplementary material for: Chromosome-scale genome assembly of Prunus pusilliflora provides novel insights into genome evolution, disease resistance, and dormancy release in Cerasus L
Source: Hortic Res. 2023 Apr 10;10(5):uhad062. doi: 10.1093/hr/uhad062 (PMC10200261; doi:10.1093/hr/uhad062)
Supplement: Web_Material_uhad062 [file web_material_uhad062.zip › Table S1.docx]

**Table S1. Phenotypic characteristics of flower and fruit organs in *Prunus pusilliflora*.**

| No. | Stamen number | Pedicel length(cm) | Flower number of each inflorescence | Total soluble solids (%) | Vertical diameter (mm) | Transverse diameter (broadside) (mm) | Transverse diameter (narrow side) (mm) | Single fruit weight (g) |
| --- | --- | --- | --- | --- | --- | --- | --- | --- |
| 1 | 35 | 0.8 | 6 | 18.2 | 10.27 | 8.39 | 7.90 | 0.51 |
| 2 | 33 | 0.9 | 7 | 14.6 | 9.25 | 7.24 | 6.42 | 0.4 |
| 3 | 34 | 0.7 | 5 | 16.0 | 10.21 | 8.08 | 7.91 | 0.31 |
| 4 | 34 | 0.9 | 5 | 15.0 | 9.03 | 7.44 | 6.76 | 0.39 |
| 5 | 33 | 0.9 | 4 | 14.2 | 9.63 | 8.01 | 7.66 | 0.41 |
| 6 | 35 | 0.7 | 5 | 16.6 | 9.07 | 7.21 | 6.38 | 0.45 |
| 7 | 36 | 0.8 | 4 | 18.0 | 9.08 | 7.86 | 6.79 | 0.32 |
| 8 | 36 | 1.0 | 5 | 15.6 | 9.05 | 6.95 | 6.59 | 0.39 |
| 9 | 35 | 1.0 | 5 | 14.0 | 9.51 | 8.05 | 6.58 | 0.39 |
| 10 | 35 | 0.9 | 6 | 15.0 | 8.64 | 7.29 | 5.98 | 0.4 |
| 11 | 36 | 0.7 | 6 | 16.6 | 8.68 | 7.3 | 6.8 | 0.41 |
| 12 | 32 | 0.9 | 3 | 18.2 | 9.28 | 7.79 | 7.25 | 0.36 |
| 13 | 30 | 0.7 | 4 | 14.6 | 9.03 | 7.63 | 6.91 | 0.35 |
| 14 | 38 | 0.7 | 6 | 16.8 | 9.1 | 7.51 | 7.32 | 0.36 |
| 15 | 36 | 0.6 | 6 | 17.0 | 8.48 | 7.18 | 7.01 | 0.39 |
| 16 | 36 | 0.7 | 6 | 19.0 | 8.46 | 7.09 | 6.6 | 0.42 |
| 17 | 39 | 0.7 | 6 | 19.0 | 9.23 | 7.68 | 7.34 | 0.47 |
| 18 | 36 | 0.9 | 6 | 17.0 | 9.15 | 7.06 | 6.48 | 0.44 |
| 19 | 40 | 0.7 | 4 | 13.5 | 8.76 | 7.16 | 6.64 | 0.39 |
| 20 | 37 | 0.9 | 5 | 16.0 | 9.26 | 5.96 | 7.02 | 0.36 |
| 21 | 37 | 1.0 | 7 | - | - | - | - | - |
| 22 | 37 | 0.9 | 6 | - | - | - | - | - |
| 23 | 34 | 0.7 | 6 | - | - | - | - | - |
| 24 | 35 | 0.8 | 6 | - | - | - | - | - |
| 25 | 37 | 0.9 | 6 | - | - | - | - | - |
| 26 | 35 | 0.7 | 7 | - | - | - | - | - |
| 27 | 38 | 0.8 | 7 | - | - | - | - | - |
| 28 | 37 | 0.8 | 8 | - | - | - | - | - |
| 29 | 32 | 0.9 | 8 | - | - | - | - | - |
| 30 | 32 | 0.9 | 6 | - | - | - | - | - |
| 31 | 35 | 1.0 | 6 | - | - | - | - | - |
| 32 | 36 | 0.8 | 6 | - | - | - | - | - |
| 33 | 36 | 0.7 | 5 | - | - | - | - | - |
| 34 | 36 | 1.0 | 6 | - | - | - | - | - |
| 35 | 34 | 0.8 | 7 | - | - | - | - | - |
| 36 | 31 | 0.6 | 7 | - | - | - | - | - |
| 37 | 34 | 0.7 | 7 | - | - | - | - | - |
| 38 | 33 | 0.8 | 6 | - | - | - | - | - |
| 39 | 34 | 0.8 | 5 | - | - | - | - | - |
| 40 | 36 | 0.6 | 6 | - | - | - | - | - |
| 41 | 41 | 1.1 | 7 | - | - | - | - | - |
| 42 | 33 | 0.8 | 6 | - | - | - | - | - |
| 43 | 41 | 1.1 | 7 | - | - | - | - | - |
| 44 | 41 | 1.0 | 7 | - | - | - | - | - |
| 45 | 43 | 1.2 | 5 | - | - | - | - | - |
| 46 | 42 | 1.0 | 5 | - | - | - | - | - |
| 47 | 39 | 1.1 | 3 | - | - | - | - | - |
| 48 | 43 | 0.9 | 5 | - | - | - | - | - |
| 49 | 41 | 1.1 | 4 | - | - | - | - | - |
| 50 | 36 | 1.1 | 7 | - | - | - | - | - |
| AVG | 36.1 | 0.854 | 5.76 | 16.245 | 9.1585 | 7.444 | 6.917 | 0.396 |
