# Supplementary material for: Chromosome-scale genome assembly of Prunus pusilliflora provides novel insights into genome evolution, disease resistance, and dormancy release in Cerasus L
Source: Hortic Res. 2023 Apr 10;10(5):uhad062. doi: 10.1093/hr/uhad062 (PMC10200261; doi:10.1093/hr/uhad062)
Supplement: Web_Material_uhad062 [file web_material_uhad062.zip › Table S2.docx]

**Table S2. Genome survey of *Prunus pusilliflora* (kmer = 19).**

| property | min | max |
| --- | --- | --- |
| Homozygous(aa) | 95.65% | 99.61% |
| Heterozygous(ab) | 0.39% | 4.35% |
| Genome Haploid | 200,331,099 bp | 303,026,329 bp |
| Genome Repeat Length | 54,904,567 bp | 83,050,157 bp |
| Genome Unique Length | 145,426,532 bp | 219,976,172 bp |
| Model Fit | 60.90% | 87.48% |
| Read Error Rate | 0.64% | 0.64% |
