# Supplementary material for: Chromosome-scale genome assembly of Prunus pusilliflora provides novel insights into genome evolution, disease resistance, and dormancy release in Cerasus L
Source: Hortic Res. 2023 Apr 10;10(5):uhad062. doi: 10.1093/hr/uhad062 (PMC10200261; doi:10.1093/hr/uhad062)
Supplement: Web_Material_uhad062 [file web_material_uhad062.zip › Table S3-S7.docx]

**Table S3. Data statistics of whole-genome sequencing for *P. pusilliflora*.**

| libraries | Total data (bp) | Read length (bp) | N50 (bp) | Coverage fold |
| --- | --- | --- | --- | --- |
| Illumina | 67,721,707,200 | 150 | - | 218.7 |
| Nanopore | 93,347,050,996 | - | 29,961 | 301.5 |
| Hi-C | 87,878,137,442 | 150 | - | 283.8 |
| Total | 248,946,895,638 | - | - | - |

**Table S4. Statistics for the *P. pusilliflora* assembly.**

| Sample ID | Length (bp) | | number | |  |
| --- | --- | --- | --- | --- | --- |
|  | Contig | Scaffold | Contig | Scaffold | GC content (%) |
| Total | 309,530,411 | 309,624,911 | 265 | 76 | 38.02 |
| Max | 24,643,276 | 59,339,823 | - | - | - |
| N50 | 6,002,992 | 33,870,278 | 13 | 4 | - |

Note: N50 refers to the size above which 50% of the total length of the sequence assembly can be found.

**Table S5. Data statistics of ordering and orienting the scaffolds on 8 pseudomolecules.**

| Chromosome | Length (bp) | N_base | Gap_ratio (%) |
| --- | --- | --- | --- |
| Chr1 | 59,339,823 | 10000 | 0.02 |
| Chr2 | 40,916,735 | 6500 | 0.02 |
| Chr3 | 36,962,455 | 2500 | 0.01 |
| Chr4 | 33,870,278 | 8500 | 0.03 |
| Chr5 | 32,235,019 | 3000 | 0.01 |
| Chr6 | 30,506,433 | 8500 | 0.02 |
| Chr7 | 30,488,219 | 4500 | 0.01 |
| Chr8 | 25,909,772 | 7500 | 0.02 |
| Total | 290,228,734 | 94500 | 0.03 |
| Anchored ratio (%) | 93.74 | - | - |
| Unanchored | 19,396,177 | - | - |
| GC content (%) | 38.02 |  |  |

**Table S6. Coverage statistics of the *P. pusilliflora* genome.**

| Total reads | Map reads | Mapping rate (%) | Properly mapping rate (%) |
| --- | --- | --- | --- |
| 469,409,152 | 454,053,561 | 96.73% | 82.18% |

**Table S7. Completeness of the assembly and the annotated genes measured by Benchmarking Universal Single-Copy Orthologs (BUSCO).**

| Description | Assembly | | Annotated genes | |
| --- | --- | --- | --- | --- |
|  | Number | Percentage (%) | Number | Percentage (%) |
| Complete BUSCOs | 1587 | 98.3 | 1562 | 96.2 |
| Complete and single-copy BUSCOs | 1511 | 93.6 | 660 | 91.4 |
| Complete and duplicated BUSCOs | 76 | 4.7 | 902 | 4.8 |
| Fragmented BUSCOs | 7 | 0.4 | 20 | 1.2 |
| Missing BUSCOs | 20 | 1.3 | 32 | 2.6 |
| Total BUSCO groups searched | 1614 | - | 1614 | - |
