# Supplementary material for: Chromosome-scale genome assembly of Prunus pusilliflora provides novel insights into genome evolution, disease resistance, and dormancy release in Cerasus L
Source: Hortic Res. 2023 Apr 10;10(5):uhad062. doi: 10.1093/hr/uhad062 (PMC10200261; doi:10.1093/hr/uhad062)
Supplement: Web_Material_uhad062 [file web_material_uhad062.zip › Table S8.docx]

**Table S8**. **Long-terminal-repeat retrotransposon assembly index (LAI) analysis and contig N50s of different genome assemblies in *Prunus* species.**

| Species | raw LAI | LAI | Contig N50 (Mb) |
| --- | --- | --- | --- |
| *Prunus pusilliflora* | 18.30 | 17.35 | 6.00 |
| *P. avium*^1^ | 24.45 | 19.68 | 3.25 |
| *P. persica*^2^ | 18.86 | 18.79 | 0.25 |
| *P. armeniaca*^3^ | 14.57 | 16.29 | 1.02 |
| *P. dulcis*^4^ | - | - | 0.10 |
| *P. serrulata*^5^ | - | - | 1.56 |
| *P. yedoensis*^6^ | 12.50 | 6.87 | 0.92 |
| *P. domestica*^7^ | 7.90 | 2.27 | 1.74 |

**References:**

1. Wang J, Liu W, Zhu D *et al.* Chromosome-scale genome assembly of sweet cherry (*Prunus avium* L.) cv. Tieton obtained using long-read and Hi-C sequencing. *Hortic Res.* 2020; **7**:122.
2. Verde I, Jenkins J, Dondini L *et al*. The Peach v2.0 release: high-resolution linkage mapping and deep resequencing improve chromosome-scale assembly and contiguity. *BMC Genomics*. 2017; **18(1)**:225.
3. Jiang F, Zhang J, Wang S *et al*. The apricot (*Prunus armeniaca* L.) genome elucidates Rosaceae evolution and beta-carotenoid synthesis. Horticulture research. 2019; **6(1)**:128.
4. Alioto T, Alexiou KG, Bardil A *et al*. Transposons played a major role in the diversification between the closely related almond and peach genomes: results from the almond genome sequence. *Plant J.* 2020; **101**: 455–72.
5. Yi XG, Yu XQ, Chen J *et al*. The genome of Chinese flowering cherry (*Cerasus serrulata*) provides new insights into cerasus species. *Hortic Res.* 2020; **7**:165.
6. Baek S, Choi K, Kim GB *et al*. Draft genome sequence of wild *Prunus yedoensis* reveals massive inter-specific hybridization between sympatric flowering cherries. *Genome Biol.* 2018; **19**:1–17.
7. Zhebentyayeva T, Shankar V, Scorza R *et al*. Genetic characterization of worldwide *Prunus domestica* (plum) germplasm using sequence-based genotyping. *Hortic Res.* 2019; **6**:12.
