# Supplementary material for: Chromosome-scale genome assembly of Prunus pusilliflora provides novel insights into genome evolution, disease resistance, and dormancy release in Cerasus L
Source: Hortic Res. 2023 Apr 10;10(5):uhad062. doi: 10.1093/hr/uhad062 (PMC10200261; doi:10.1093/hr/uhad062)
Supplement: Web_Material_uhad062 [file web_material_uhad062.zip › Table S9-S10.docx]

**Table S9. Repetitive elements predicted from the *P. pusilliflora* assembly.**

| Type | Length (bp) | Rate (%) |
| --- | --- | --- |
| Class I: Retrotransposon | 83,976,806 | 28.93 |
| Class II: DNA Transposon | 37,275,546 | 12.84 |
| Low Complexity | 18,698 | 0.01 |
| Tandem repeat | 6,345,819 | 2.19 |
| Unclassified | 14,821,827 | 5.11 |
| Total content | 142,438,696 | 49.08 |

**Table S10. Summary of transposable elements in the *P. pusilliflora* genome*.***

| Type | Length (bp) | Rate (%) |
| --- | --- | --- |
| LTR/*Ty1_Copia* | 32,463,134 | 11.19 |
| LTR/*Ty3_Gypsy* | 45,577,864 | 15.70 |
| LTR-other | 2,171,434 | 0.75 |
| SINE | 30,251 | 0.01 |
| LINE | 3,734,123 | 1.29 |
| EnSpm/CACTA | 17,494,997 | 6.03 |
| Harbinger | 4,311,482 | 1.49 |
| Helitron | 2,919,759 | 1.01 |
| MuDR | 3,432,894 | 1.18 |
| Tcl/Mariner | 178,623 | 0.06 |
| hAT | 7,614,739 | 2.62 |
| DNA-other | 1,323,052 | 0.46 |
| Total | 121,252,352 | 41.79 |
