# Supplementary material for: Chromosome-scale genome assembly of Prunus pusilliflora provides novel insights into genome evolution, disease resistance, and dormancy release in Cerasus L
Source: Hortic Res. 2023 Apr 10;10(5):uhad062. doi: 10.1093/hr/uhad062 (PMC10200261; doi:10.1093/hr/uhad062)
Supplement: Web_Material_uhad062 [file web_material_uhad062.zip › Table S11-S12.docx]

**Table S11. Comparison of gene space between *P. pusilliflora* and other species.**

| Species | Gene number | Average gene length (bp) | Average exon  Length (bp) | Average CDS length (bp) | Average exon number |
| --- | --- | --- | --- | --- | --- |
| *Arabidopsis thaliana* | 27,444 | 2,341 | 237 | 1,221 | 5 |
| *P. armeniaca* | 37,521 | 2,563 | 267 | 1,243 | 5 |
| *P. avium* | 38,277 | 2,674 | 241 | 1,075 | 4 |
| *P. persica* | 23,050 | 3,605 | 259 | 1,392 | 5 |
| *P. dulcis* | 23,065 | 3,498 | 257 | 1,383 | 5 |
| *P. persica* | 29,094 | 3,600 | 238 | 1,285 | 5 |
| *Rosa chinensis* | 30,924 | 3,604 | 270 | 1,340 | 5 |
| *P. yedoensis* | 41,294 | 2,155 | 221 | 954 | 4 |
| *P. pusilliflora* | **33,035** | 2,828 | 233 | 1,071 | 5 |

**Table S12. Functional annotation of the predicted genes.**

| Database | Annotated Number | Annotated Percent (%) |
| --- | --- | --- |
| NR | 32,454 | 98.24 |
| Swiss-Prot | 22,172 | 67.12 |
| TAIR | 25,162 | 76.17 |
| MSU | 25965 | 78.60 |
| GO | 10,174 | 30.80 |
| KEGG | 12,888 | 39.01 |
| COG | 27,458 | 83.12 |
| eggNOG | 27,458 | 83.12 |
| Pfam | 21,905 | 66.31 |
| Annotated | 32,463 | 98.27 |
| Total | 33,035 | - |
