# Supplementary material for: Chromosome-scale genome assembly of Prunus pusilliflora provides novel insights into genome evolution, disease resistance, and dormancy release in Cerasus L
Source: Hortic Res. 2023 Apr 10;10(5):uhad062. doi: 10.1093/hr/uhad062 (PMC10200261; doi:10.1093/hr/uhad062)
Supplement: Web_Material_uhad062 [file web_material_uhad062.zip › Table S14.docx]

**Table S14. Non-coding RNAs predicted from the *P. pusilliflora* genome.**

| Type | | Copy number | Average length (bp) | Total length (bp) |
| --- | --- | --- | --- | --- |
| miRNA | | 149 | 122 | 18,189 |
| tRNA | | 756 | 75 | 56,390 |
| rRNA | 18S | 189 | 1,037 | 195,933 |
|  | 28S | 356 | 113 | 40,242 |
|  | 5.8S | 104 | 107 | 11,164 |
|  | 5S | 598 | 90 | 53,983 |
|  | total | 1,247 | 242 | 301,322 |
| snRNA | CD-box | 156 | 104 | 16,298 |
|  | HACA-box | 18 | 118 | 2,120 |
|  | splicing | 102 | 147 | 14,985 |
|  | total | 276 | 121 | 33,403 |
