# Supplementary material for: Chromosome-scale genome assembly of Prunus pusilliflora provides novel insights into genome evolution, disease resistance, and dormancy release in Cerasus L
Source: Hortic Res. 2023 Apr 10;10(5):uhad062. doi: 10.1093/hr/uhad062 (PMC10200261; doi:10.1093/hr/uhad062)
Supplement: Web_Material_uhad062 [file web_material_uhad062.zip › Table S18.docx]

**Table S18. The statistics of *Prunus pusilliflora vs. P. avium*, *P. pusilliflora vs*. *P. persica*, and *P. pusilliflora vs*. *P. serrulata* synteny maps.**

| Type | *P. pusilliflora vs*. *P. avium* | *P. pusilliflora vs*. *P. persica* | *P. pusilliflora vs*. *P. serrulata* |
| --- | --- | --- | --- |
| Total synteny block (number) | 2803 | 2576 | 3778 |
| Block on the same chromosome (number) | 2722 | 2552 | 3645 |
| Block on the different chromosome (number) | 81 | 24 | 133 |
| Block on the same direction (number) | 2535 | 2542 | 3611 |
| Block on the opposite orientations (number) | 268 | 36 | 167 |
