# Supplementary material for: Chromosome-scale genome assembly of Prunus pusilliflora provides novel insights into genome evolution, disease resistance, and dormancy release in Cerasus L
Source: Hortic Res. 2023 Apr 10;10(5):uhad062. doi: 10.1093/hr/uhad062 (PMC10200261; doi:10.1093/hr/uhad062)
Supplement: Web_Material_uhad062 [file web_material_uhad062.zip › Table S41.pdf]

Table S41. Chromosome distribution of different categories of RGAs in *P. pusilliflora*.

**Categorie** / **Chr**	NBS	RLK	RLP	RPW8	TM-CC	**Total**
chr1	60	97	16	1	24	**198**
chr2	96	62	30	10	17	**215**
chr3	25	73	6	0	15	**119**
chr4	9	133	23	3	8	**176**
chr5	38	41	2	0	26	**107**
chr6	21	98	14	0	16	**149**
chr7	44	46	7	8	17	**122**
chr8	80	36	15	1	17	**149**
un_anch	31	46	13		5	**95**
**Total**	**404**	**632**	**126**	**23**	**145**	**1330**
