# Supplementary material for: Chromosome-scale genome assembly of Prunus pusilliflora provides novel insights into genome evolution, disease resistance, and dormancy release in Cerasus L
Source: Hortic Res. 2023 Apr 10;10(5):uhad062. doi: 10.1093/hr/uhad062 (PMC10200261; doi:10.1093/hr/uhad062)
Supplement: Web_Material_uhad062 [file web_material_uhad062.zip › Table S42.pdf]

Table S42. Chromosome distribution of different categories of NBS-type R genes in *P. pusilliflora*.

**Categories** / **Chr**	CN	CNL	NBS	NL	others	RN	RNL	TN	TNL	TX	**total**
chr1	6	3	12	15	2	0	1	6	13	2	**60**
chr2	7	10	7	22	1	0	0	16	27	6	**96**
chr3	1	3	10	5	0	0	0	1	5	2	**27**
chr4	1	1	1	2	0	0	1	0	1	2	**9**
chr5	0	0	7	9	0	1	0	9	10	0	**36**
chr6	2	2	5	8	0	1	0	3	0	0	**21**
chr7	3	1	10	4	0	8	9	3	3	3	**44**
chr8	2	6	7	23	8	0	0	13	19	2	**80**
un_anch	5	3	6	7	0	3	0	2	3	2	**31**
**total**	**27**	**29**	**65**	**95**	**11**	**13**	**11**	**53**	**81**	**19**	**404**
