# Supplementary material for: Chromosome-scale genome assembly of Prunus pusilliflora provides novel insights into genome evolution, disease resistance, and dormancy release in Cerasus L
Source: Hortic Res. 2023 Apr 10;10(5):uhad062. doi: 10.1093/hr/uhad062 (PMC10200261; doi:10.1093/hr/uhad062)
Supplement: Web_Material_uhad062 [file web_material_uhad062.zip › Table S45.pdf]

Table S45. Chromosome distribution of different categories of NBS-type R genes in *P. avium*.

**Categories** / **Chr**	CN-type	CNL-type	NBS-type	NL-type	RNL-type	RN-type	TN-type	TNL-type	TX-type	others	Total
chr1	4	11	12	9	1	0	0	3	5	0	**45**
chr2	5	37	5	13	1	0	0	4	3	1	**69**
chr3	1	9	10	4	0	0	0	2	0	0	**26**
chr4	0	4	3	4	0	0	0	0	2	0	**13**
chr5	0	0	0	10	1	1	2	2	1	0	**17**
chr6	0	2	4	12	1	0	1	0	0	0	**20**
chr7	1	6	2	4	11	3	1	0	0	1	**29**
chr8	2	13	7	22	0	0	1	6	2	5	**58**
**Total**	**13**	**82**	**43**	**78**	**15**	**4**	**5**	**17**	**13**	**7**	**277**
