# Supplementary material for: Chromosome-scale genome assembly of Prunus pusilliflora provides novel insights into genome evolution, disease resistance, and dormancy release in Cerasus L
Source: Hortic Res. 2023 Apr 10;10(5):uhad062. doi: 10.1093/hr/uhad062 (PMC10200261; doi:10.1093/hr/uhad062)
Supplement: Web_Material_uhad062 [file web_material_uhad062.zip › Table S50.docx]

**Table S50. BUSCO analyses of genomes for several *Prunus* species and *Rosa chinensis*.**

| Type | C^*^ | S^*^ | D^*^ | F^*^ | M^*^ | n^*^ |
| --- | --- | --- | --- | --- | --- | --- |
| *P.armeniaca*^1^ | 97.40% | 95.00% | 2.40% | 0.60% | 2.00% | 1614 |
| *P.avium*^2^ | 98.30% | 95.50% | 2.80% | 0.40% | 1.30% | 1614 |
| *P.dulcis*^3^ | 97.70% | 93.40% | 4.30% | 0.20% | 2.10% | 1614 |
| *P.persica*^4^ | 99.30% | 97.40% | 1.90% | 0.10% | 0.60% | 1614 |
| *P.pusilliflora* | 98.30% | 93.60% | 4.70% | 0.40% | 1.30% | 1614 |
| *P.serrulata*^5^ | 97.70% | 92.20% | 5.50% | 0.80% | 1.50% | 1614 |
| *Rosa chinensis*^6^ | 98.60% | 94.60% | 4.00% | 0.50% | 0.90% | 1614 |

Note: *

C: Complete BUSCOs

S: Complete and single-copy BUSCOs

D: Complete and duplicated BUSCOs

F: Fragmented BUSCOs

M: Missing BUSCOs

n: Total BUSCO groups searched

**References：**

1. Jiang F, Zhang J, Wang S, Yang L, Luo Y, Gao S, et al. The apricot (*Prunus armeniaca* L.) genome elucidates Rosaceae evolution and beta-carotenoid synthesis. *Horticulture research*. 2019; **6(1)**:128.

2. Wang J, Liu W, Zhu D *et al.* Chromosome-scale genome assembly of sweet cherry (*Prunus avium* L.) cv. Tieton obtained using long-read and Hi-C sequencing. *Hortic Res.* 2020; **7**:122.

3. Alioto T, Alexiou KG, Bardil A *et al*. Transposons played a major role in the diversification between the closely related almond and peach genomes: results from the almond genome sequence. *Plant J.* 2020; **101**: 455–72.

4. Verde I, Jenkins J, Dondini L *et al*. The Peach v2.0 release: high-resolution linkage mapping and deep resequencing improve chromosome-scale assembly and contiguity. *BMC Genomics*. 2017; **18(1)**:225.

5. Yi XG, Yu XQ, Chen J *et al*. The genome of Chinese flowering cherry (*Cerasus serrulata*) provides new insights into cerasus species. *Hortic Res.* 2020; **7**:165.

6. Raymond O, Gouzy J, Just J *et al*. The *Rosa* genome provides new insights into the domestication of modern roses. *Nature Genetics*. 2018; **50**:772–7.

.
