# Supplementary material for: Chromosome-scale genome assembly of Prunus pusilliflora provides novel insights into genome evolution, disease resistance, and dormancy release in Cerasus L
Source: Hortic Res. 2023 Apr 10;10(5):uhad062. doi: 10.1093/hr/uhad062 (PMC10200261; doi:10.1093/hr/uhad062)
Supplement: Web_Material_uhad062 [file web_material_uhad062.zip › Table S51.docx]

**Table S51 Sequence of primers used for** **qRT-PCR in this study.**

| **Gene Name** | **Forward primer sequences (5’→3’)** | **Reverse primer sequences (5’→3’)** |
| --- | --- | --- |
| *Pavi-Actin* | CGGTATTGCAGACGGATGAGC | GGTACTGAGGGATGCAAGGATGG |
| *Ppus-Actin* | CGGTATTGCAGACGGATGAGC | GGTACTGAGGGATGCAAGGATGG |
| *Cv-GAPDH* | ATGGCTCCCATCAAGGTCG | GGGTGGAGTCGTACTTGAGCATGT |
| *Pc-Actin* | AGGAGATGGCCAAGTTAGC | CCGACTCATCATACTCGG |
| *Pst-Gap-1* | TTGACCAGAATCTGCACCCC | CTTTGGCGGGAGAGACTTCA |
